# Identification of H1N2 influenza viruses in turkeys after spillover from swine and *in vitro* characterization

**DOI:** 10.1016/j.virusres.2025.199634

**Published:** 2025-09-21

**Authors:** Chloé Chavoix, Pascale Massin, François-Xavier Briand, Katell Louboutin, Rachel Busson, Florent Souchaud, Gautier Richard, Claire Martenot, Aurélie Le Roux, Edouard Hirchaud, Yannick Blanchard, Sophie Le Bouquin-Leneveu, Axelle Scoizec, Céline Deblanc, Séverine Hervé, Audrey Schmitz, Eric Niqueux, Gaëlle Simon, Ronan Le Goffic, Béatrice Grasland

**Affiliations:** a Anses, Ploufragan-Plouzané-Niort laboratory, Unit Virology, Immunology and Parasitology in Poultry and Rabbits, BP 53, 31 rue des fusillés Ploufragan, 22440, France; b Anses, Ploufragan-Plouzané-Niort laboratory, Unit Swine Virology, Innovation and Genomics, Ploufragan, France; c Anses, Ploufragan-Plouzané-Niort laboratory, Unit Epidemiology, Health and Welfare, Ploufragan, France; d INRAE, UMR892 VIM, Jouy-en-Josas, France

**Keywords:** Influenza A virus, H1N2, Cross-species transmission, Swine, Turkey, Molecular markers, Cell and egg infection

## Abstract

Influenza A viruses (IAVs) circulate among animals and humans and can cross species barriers to adapt to new hosts. In France, since 2020, a new genotype named H1_av_N2#E has predominated in pig farms. In parallel, this virus was detected in 19 breeding turkey flocks and in a human case, indicating interspecies transmission. The objectives of this study were (i) to analyze viral sequences detected in turkeys between April 2020 and January 2023 and compare them with swine sequences to identify potential markers of adaptation and (ii) to characterize three representative viruses *in vitro* and *in ovo* on MDCK, MLE15 and A549 cells, as well as on embryonated chicken eggs. Results suggest that following initial swine-to-turkey spillovers, the virus circulated between turkey farms. Phylogenetic analyses and host origin guided the selection of three viruses: a reference swine virus (A), a turkey strain closely related to swine viruses (B) and a virus apparently circulating in turkeys (C). Three molecular markers may contribute to turkey adaptation: the E233K, under positive selection pressure, or E236K mutations in the 220-loop of the receptor-binding site of HA protein and two NA substitutions, T401N in antigenic site 2d and S416N. Replication kinetics showed that at low MOI (0.001), virus C produced more infectious particles on MDCK and A549 cells, whereas at high MOI (0.1), virus B produced more. *In ovo*, infectious particles were generated for viruses A and B but not efficiently for virus C, in contrast to mammalian cells where production was higher.

## Highlights

• Transmissions of a new H1N2 IAV from swine to turkey occurred in France.
• This virus might have been transmitted between turkey farms.
• E233K or E236K on HA, T401N and S416N on NA may be markers of adaptation in turkeys.
• Viruses showed slight differences across mammalian-origin cell lines.
• Eggs allowed more discrimination between viruses but were less efficient than cells.

## 1 Introduction

Influenza A viruses (IAVs) are enveloped viruses from the Orthomyxoviridae family, with eight single-stranded RNA segments of negative polarity. The two main surface glycoproteins are the hemagglutinin (HA) and the neuraminidase (NA) which define the H_x_N_y_ subtypes of IAVs. Influenza viruses evolve through two main mechanisms: point mutations during replication and genome segment reassortments between different strains. When these changes affect antigenic sites on hemagglutinin and neuraminidase, they lead to antigenic drift and antigenic shift (Webster et al. 1982). These antigenic changes allow the virus to escape immune recognition and contribute to its continued circulation in human and animal populations (Webster and Govorkova 2014).

The waterfowl, particularly anseriforms and charadriiforms, are considered to be the main reservoirs of IAVs genetic diversity with the detection of 16 HA and 9 NA subtypes, in addition to H19. IAVs can cross the species barrier and infect a wide range of host species (Long et al. 2019). It has been demonstrated several times between birds and swine (Yassine, Lee, and Saif 2011) but also between other species including mammal species, such as marine mammals (Fereidouni et al. 2016) or dairy cattle (Burrough et al. 2024). The phenomenon of reassortment, along with the ability of IAVs to cross the species barrier and subsequently spread, represents a major public health concern. This has been clearly described with the four major flu pandemics of the 20^th^ and 21^st^ centuries (1918, 1957, 1968, and 2009); each caused by the emergence of novel IAV strain in humans (Horimoto and Kawaoka 2005; Smith et al. 2009). Interspecies transmissions of IAVs from swine to turkeys are already described events, with several cases described (Pillai et al. 2010). First evidence of turkey infections with swine IAVs was described in the United States of America (USA) in 1975 (Mohan et al. 1981). Other transmissions of swine IAVs have been reported in several countries such as in the USA with a H1N2 subtype (Suarez et al. 2002), in the USA and Canada with a H3N2 subtype (Choi et al. 2004) and in Chile with a H1N1 subtype (Mathieu et al. 2010). In Europe, since late 80’s, transmission events of swine H1N1 virus have also been reported, and they mainly involve “Eurasian avian-like swine H1N1” (Ludwig et al. 1994). In France, the first case of turkey infection with a swine IAV was detected in 1985 with a H1N1 subtype (Andral et al. 1985). All these species barrier-crossing events illustrate that IAVs transmissions between avian, swine and humans are a threat for public health.

Despite these spillovers of IAVs, host range restriction exists and limits this interspecies transmission. One of the initial barriers to host infection by an IAV is the virus’s ability to bind to specific cellular receptors. Indeed, the HA glycoprotein of IAV recognizes and binds to sialic acids (SA) present on the cell surface. It has been demonstrated that the nature of the linkage between SA and the carbohydrate chains on glycoproteins and glycolipids of the cell surface is determinant for the virus host specificity (Stencel-Baerenwald et al. 2014). In birds such as chickens and ducks, α-2,3-linked SA are predominant in the upper respiratory tract. In swine, α-2,6-linked SA are predominant in the trachea and pulmonary alveoli, but α-2,3-linked SA are also present in the nasal mucosa. This distribution of both SA receptor types along the respiratory tract, similar to that in humans, makes pigs considered as “mixing vessels” (Nelli et al. 2010; Kristensen et al. 2024). Turkeys also possess receptors for both avian and human/swine IAVs in their respiratory system (Kimble, Nieto, and Perez 2010). Pigs and turkeys, as well as humans, can therefore be considered as important intermediaries, capable of making possible reassortments between swine, avian, and human IAVs.

Beyond the viral receptor, other host restriction factors influence cross-species transmission. Host body temperature differs between species. Consequently, the temperatures at the sites of viral replication also vary (33°C and 37°C in the upper respiratory tract of humans and pigs respectively and close to 40°C in birds such as chickens and ducks) and can affect viral replication (Massin et al. 2010). Viral proteins, such as those of the polymerase complex, also depend on host-specific cellular cofactors. A key example is the acidic nuclear phosphoprotein 32 (ANP32) family, where mammalian and avian isoforms differ in their ability to support polymerase activity (Peacock et al. 2023). The host’s immune response further contributes to restriction, particularly through interferon-stimulated genes such as the myxovirus resistance (Mx) protein, which has been extensively studied in mammalian models including humans and mice (Dittmann et al. 2008). These restriction factors, although varying between species, therefore limit the transmission of IAVs from one host to another.

In swine, the three main subtypes of IAVs circulating in Europe and France are H1N1, H1N2 and H3N2 (Watson et al. 2015; Richard, Herve, et al. 2025). In 2016, a classification based on the H1 hemagglutinin sequences from swine IAVs has been proposed (Anderson et al. 2016). Three major lineages have been defined: lineage 1A “Classical swine lineage” (H1_sw_) includes viruses related to those of the 1918 H1N1 Spanish flu pandemic; lineage 1B, “Human seasonal lineage” (H1_hu_), groups viruses from human-to-swine seasonal influenza transmissions and lineage 1C, “Eurasian avian like” (H1_av_), groups viruses from the introduction of an avian H1 virus from wild birds to swine. The lineage 1C is divided into seven distinct genetic clades: 1C.2.1, 1C.2.2, 1C2.3, 1C2.4.1, 1C2.4.2, 1C2.4.3 and 1C2.5 (WOAH 2024) and is currently the predominant lineage circulating in Europe (Richard, Ryt-Hansen, et al. 2025).

In France, between 2009 and 2019, several IAV subtypes circulated in pig populations, mainly H1_av_N1, H1_hu_N2, and sporadically H3N2 (Chauhan and Gordon 2020). The pandemic H1N1_pdm_09 subtype was also introduced from humans in 2009 (Smith et al. 2009). Since 2020, a new genotype of swine IAV named H1_av_N2#E was detected in pig farms in Brittany (France) and then quickly became the predominant genotype circulating in pig farms in France (Richard, Herve, et al. 2025; Hervé et al. 2021). The infected pigs were mainly growing pigs (80.2 %) and breeding pigs (19.8 %) which exhibited flu-like symptoms of moderate to severe intensity (Hervé et al. 2021). As described by Richard and coll., phylogenetic analyses allowed to show that the H1_av_N2#E genotype possesses an HA gene classified within HA-Clade 1C.2.4 among the 1C EA lineage by OFFLU (WOAH 2021; Richard, Herve, et al. 2025). This virus was probably introduced *in toto* in 2015 from Denmark to the South-West of France where two cases were detected four months apart. No other cases were detected in France until 2020. This virus was likely reintroduced into France in late 2019, probably from Denmark, before spreading widely from 2020. All the strains detected since 2020 have the particularity of exhibiting a double deletion of three amino acids in the HA gene, at position 137, and at positions 146–147 within the SA receptor binding site (RBS). Simultaneously to its detection from swine in Brittany, this virus was also detected in breeding turkey farms in the same area. In France, data from annual serological surveillance of breeding and fattening turkey flocks indicate that IAVs circulate only sporadically in these populations (Anses 2010, 2021). Occasional detections of various subtypes have been reported for these last 25 years, including both low pathogenic (H5N3, H6N2, H6N1, H5N1, H1N2) and highly pathogenic strains (H5N1) (Personal communication, Anses Avian Influenza French National Reference Laboratory (AI French NRL)) (Corrand et al. 2012). In September 2021, a new case of cross-species transmission of this virus, this time from swine to humans, occurred in Brittany. The patient was admitted to intensive care for a severe lower acute respiratory infection. The man had been in contact with live pigs a few days before developing symptoms, and he was diagnosed with the same swine H1N2 virus. This clinical case sparked concern within the community, although no human-to-human transmission has been detected, and the patient has since recovered (Simon et al. 2022).

The detection of the genotype H1_av_N2#E in swine, turkeys and in human raises the question of a species barrier crossing of this virus from swine to turkey, from swine to human and its adaptation to each species. The first objective of this study was to analyze the sequences detected in turkeys and to compare them to swine sequences in order to identify potential molecular markers of adaptation to the turkey host. The second objective was to characterize *in vitro* two viruses isolated from turkeys, selected based on their phylogenetic features, along with one swine-origin virus previously described, on different cell types.

## 2 Material and methods

### 2.1 Viral sequences

Swine H1_av_N2#E viral sequences were obtained by the swine influenza French NRL (Anses Ploufragan-Plouzané-Niort) and submitted in Genbank as already described (Richard, Herve, et al. 2025). Turkey samples were collected on farms as part of event-based surveillance (Briand et al. 2017). RNA extracts from M-positive samples were sent to the AI French NRL (Anses Ploufragan-Plouzané-Niort). Turkey H1_av_N2#E viral genomes were fully sequenced by Next-Generation Sequencing (NGS). Amplifications of the 8 viral genomic segments prior to sequencing was performed with universal IAV primers and using the SuperScript III one-step RT-PCR system (Thermo Fisher Scientific, Waltham, MA, USA) (B. Zhou et al. 2009). Libraries and complete genome sequencing were carried out using the Ion Proton instrument (Thermo Fisher, Carlsbad, *CA*, USA) that allowed the determination of a consensus sequence for each virus as previously described (Briand et al. 2017). From April 2020 to January 2023, 19 turkey sequences were obtained and submitted in GISAID database. Accession numbers and collection dates are reported in Supplementary Table 1.

### 2.2 Phylogenetic and antigenic analyses

Nucleotide sequences of the eight segments from French swine and turkey H1_av_N2#E were concatenated and aligned using the MAFFT software (version 7.5.0) (Katoh and Standley 2013) with default parameters. The phylogenetic tree was constructed using Iq-Tree software (Nguyen et al. 2015) (with 1000 ultrafastbootstrap replicates) with the Maximum Likelihood method and the GTR+F+I+R4 model (General Time Reversible + Frequency + Invariant sites + category 4 of Rate heterogeneity) corresponding to the most suitable model determined by ModelFinder (Kalyaanamoorthy et al. 2017). The two swine influenza H1_av_N2#E sequences detected in 2015 in the South-West of France, A/swine/France/64–150152/2015 and A/swine/France/65–150242/2015 (accession numbers: MT378579 to MT378586 and MT379339 to MT379346, respectively), were used as root. Individual phylogenetic trees were generated for each of the 8 segments using the same methodology as described earlier in this section, in order to verify whether the topology observed in the concatenated phylogeny was also consistent across individual segments (Supplementary Figs. 1-8). Subsequently, the sequences were translated into amino acids and compared with each other. For amino acid numbering of all segments, the first amino acid corresponded to the first coding methionine. The HA numbering of the amino acid sequences was based on the first viral sequence of influenza H1_av_N2#E detected in swine in Brittany in February 2020, A/swine/France/56–200,050/2020, (accession numbers: MZ088174 to MZ088181) (Richard, Herve, et al. 2025).

To compare amino acids composition of the 12 major proteins (PB2, PB1, PB1-F2, PA, PA-X, HA, NP, NA, M1, M2, NS1 and NS2), three groups of sequences were defined based on the phylogenetic analyses and on the host species of origin. The comparison was realized and visualized using the Ggseqlogo R package (Wagih 2017). This analysis aimed to identify positions showing notable differences among the three groups of sequences. For each position, amino acid frequencies were computed for each group based on the frequency matrices. Then, for each pair of groups *(i, j)*, the sum of absolute differences in amino acid frequencies was calculated using the following formula:

$$Sum\;of\;differences=\sum_{AA=1}^{20}\left|f\;group\;i\;\left(AA\right)-f\;group\;j\;\left(AA\right)\right|$$

where “*f group i(AA)”* and “*f group j(AA)”* represented the frequencies of a given amino acid (AA) in group i and group j, respectively. A position was considered variant if this sum was greater than 0.25 in at least one of the three pairwise comparisons, indicating a difference in residue distribution between the groups. This threshold was empirically chosen after data analysis and was based on both biological and practical considerations. It represented a compromise that allowed the identification of a limited but relevant number of specific group positions, facilitating their visual representation and biological interpretation. This analysis included the five major antigenic sites of H1 hemagglutinin (Sa, Sb, Ca1, Ca2 and Cb) (Sriwilaijaroen and Suzuki 2012) and neuraminidase (2a, 2b, 2c, 2d and 4) (Richard, Herve, et al. 2025). The three main structural elements constituting the RBS of hemagglutinin (130-loop, 190-helix, and 220-loop) (Sriwilaijaroen and Suzuki 2012) were also included in the analysis.

Potential N-glycosylation sites on HA and NA proteins were predicted using the NetNGlyc 1.0 tool (Technical University of Denmark, https://services.healthtech.dtu.dk/services/NetNGlyc-1.0/). This algorithm identifies N-X-S/T consensus motifs and evaluates the likelihood of effective glycosylation using a neural network trained on experimental data. Protein sequences of HA and NA from the three studied viruses (“Swine virus”, “Turkey swine-like virus” and “Turkey virus”), representative of the sequence groups defined above, were submitted to the server. Positions predicted as glycosylated with a score above the recommended program threshold were retained and compared with the positions described above, namely antigenic sites and positions of interest with more than 25 % differences between at least two groups.

### 2.3 Selection pressure analyses

Selection pressure analyses were performed on the 12 major proteins using Datamonkey/HyPhy (default MG94xREV model) (Kosakovsky Pond et al. 2020; Weaver et al. 2018). For each protein, four subsets were analyzed: the complete set of sequences and the three groups defined through phylogenetic analyses. Three site-by-site methods were applied. The MEME (Mixed Effects Model of Evolution) method was used to detect episodic positive selection, thereby identifying diversifying positions (Murrell et al. 2012). The FEL (Fixed Effects Likelihood) method was used to detect pervasive selection, classifying a site as diversifying if β > α and purifying if β < α, where α represented the synonymous substitution rate and β the nonsynonymous substitution rate (Kosakovsky Pond and Frost 2005). For both methods, the default significance threshold was set at p = 0.1. The SLAC (Single-Likelihood Ancestor Counting) method was used to estimate nonsynonymous (dN) and synonymous (dS) substitutions, classifying a site as diversifying if p(dN > dS) ≤ 0.1 and purifying if p(dN < dS) ≤ 0.1 (Kosakovsky Pond and Frost 2005). For each protein, subset and method, the number of purifying and diversifying sites was counted and represented graphically.

### 2.4 Cells and media

Madin-Darby Canine Kidney (MDCK), murine Mouse Lung Epithelial cells (MLE15) and human adenocarcinoma lung cells (A549) were used. All three cell types were grown at 37°C, 5 % CO2. MDCK cells were cultured in a complete medium composed of Minimum Essential Medium (MEM, Gibco, ThermoFisher Scientific, Waltham, MA, USA), supplemented with 5 % of decomplemented fetal calf serum (FCS) (Eurobio Scientific, Les Ulis, France), antibiotics (100 U/mL of penicillin and 1 mg/mL of streptomycin, Sigma-Aldrich Merck, Darmstadt, Germany), and 0.01 M of tricine (Sigma-Aldrich Merck, Darmstadt, Germany). The infection medium consisted of the complete medium without FCS but supplemented with 2 µg/mL of TPCK-treated trypsin (N-tosyl-L-phenylalanine chloromethyl ketone-treated trypsin) (Worthington Biochemical Corporation, Lakewood, NJ, USA). MLE15 and A549 cells were cultured in a complete medium, composed of MEM supplemented with 10 % of decomplemented FCS and antibiotics. The infection medium consisted of the complete medium without FCS but supplemented with TPCK-treated trypsin at concentrations of 1 µg/mL for MLE15 cells and 0.2 µg/mL for A549 cells.

### 2.5 Production of the viral strains

The swine virus, A/swine/France/35–200,154/2020 (accession numbers: MZ088854 to MZ088861) (Richard, Herve, et al. 2025), was isolated on MDCK cells by the swine influenza French NRL from nasal swab supernatant, as already described (Deblanc et al. 2024). It was subsequently propagated through two successive infections on MDCK cells. Supernatants were subjected to clarification by centrifugation to constitute the viral stock and antibiotics (20 U/mL of penicillin and 0.2 mg/mL of streptomycin) were added. The two turkey viruses, A/turkey/France/20P005076/2020 (accession numbers: EPI4543932 to EPI4543939) and A/turkey/France/21P011515/2021 (accession numbers: EPI4544020 to EPI4544027), were isolated by the avian influenza French NRL. 200 µL of swab supernatant were inoculated into 9-day-old specific-pathogen-free (SPF) embryonated chicken eggs (Anses Ploufragan-Plouzané-Niort laboratory, France). The eggs were incubated at 37 °C and candled daily. Allantoic fluids were collected either from dead embryos or after 4 days of incubation and subjected to a hemagglutination test. Allantoic fluids with positive hemagglutination test were pooled and clarified by centrifugation. Two successive infections were performed on MDCK cells. Cell supernatants were subjected to clarification by centrifugation to constitute the viral stock and antibiotics (20 U/mL of penicillin and 0.2 mg/mL of streptomycin) were added. All viral stocks were stored at -80°C until further use. All viral stocks were sequenced by NGS according to the same protocol as described in the “Viral sequences” section.

### 2.6 Viral and genomic replication kinetics on cells

For each virus, viral and genomic replication kinetics were performed on different cell types: MDCK, MLE15 and A549 cells. Cell culture plates (12-well) were seeded 24 hours before infection with 4 × 10⁵ cells per well in a volume of 1 mL of complete medium. For each virus, cells were infected in three replicates for two different multiplicity of infection (MOI) values: 0.1 and 0.001. Viruses were diluted in medium without FCS. Cells were washed twice with medium without FCS and infected with 250 µL of the diluted virus or Phosphate-Buffered Saline (PBS) (ThermoFisher Scientific, Waltham, MA, USA) for mock condition. Cells were incubated for 1 hour at 37°C, 5 % CO2, with gentle shaking every 15 min. The inoculum was removed and cells were washed once with medium without FCS. Infected cells were incubated at 37°C, 5 % CO₂ after the addition of 1 mL of infection medium. At 0, 6, 11, 24, 48 and 72 hours post-infection (hpi), the cell supernatants were collected. Viral genomic loads and infectious titers were determined as described in subsequent sections. For the graph, at each post-infection time point the value obtained at time 0 was subtracted to eliminate background noise caused by residual viral particles present at the time of infection.

### 2.7 Viral and genomic replication kinetics on embryonated eggs

For each virus, viral and genomic replication kinetics were performed on 12-day-old specific-pathogen-free (SPF) embryonated chicken eggs. For each virus and each post-infection time point, three replicates were performed. Eggs were inoculated via the allantoic route with 100 µL of virus diluted in PBS at 4 × 10^5^ TCID_50_/mL, an infectious dose corresponding to the dilution at MOI 0.1 used for viral replication kinetics on cells. At 0, 1, 6, 11, 24, 48 and 72 hpi, the eggs were placed at +4°C and allantoic fluids were collected. Viral genomic loads and infectious titers were determined as described in subsequent sections.

### 2.8 Quantification of genomic viral loads

Total RNA was extracted from samples using the King Fisher automated system and the ID Gene™ Mag Fast Extraction Kit (Innovative-Diagnostics, Grabels, France) according to manufacturer’s instructions. Viral genome was quantified using a TaqMan real-time reverse transcriptase quantitative polymerase chain reaction (rRT-qPCR) specific of the HA gene from H1_av_N2#E viruses. This rRT-qPCR, using the QuantiTect Virus Kit (Qiagen, Hilden, Germany), was used as previously described (Richard, Herve, et al. 2025). Alignment of the two turkey viral sequences with the PCR primers revealed no mismatch, suggesting that the PCR efficiency will not be compromised. Quantification was allowed by the use of a RNA transcript targeting the HA gene, produced after TOPO TA cloning by *in vitro* transcription (ThermoFisher Scientific, Waltham, MA, USA) (Green and Sambrook 2021). Viral genomic loads were expressed as the number of HA gene copies/mL of cell supernatant.

### 2.9 Quantification of infectious viral loads

Cell culture plates (96-wells) were seeded 24 hours before titration with 3×10^4^ MDCK cells per well in a volume of 200 µL of cultured medium. The sample to be titrated were serial 10-fold diluted (10^−1^ to 10^−10^) in infection medium. Cells were washed twice with medium without FCS, infected in 8 replicates per dilution and then incubated at 37°C, 5 % CO2. After 72 hours, cytopathic effects were observed and the Tissue Culture Infectious Dose for 50 % of the cells (TCID_50_) per mL of sample was calculated using the Reed and Muench formula (Reed and Muench 1938). Infectious viral loads were expressed as TCID_50_/mL of cell supernatant.

### 2.10 Statistical tests

The mean of the three replicates and the standard deviation were calculated. For each time point and between the three viruses, a non-parametric Kruskall-Wallis statistical test was performed using a Dunn’s multiple comparisons test with all three conditions compared in pairs. Significant differences were considered if *p* < 0.05. Data analyses and graphing were performed using R software (version 4.4.2) and GraphPad Prism software (version 8.0.1).

## 3 Results

### 3.1 Detection of H1_av_N2#E cases in turkeys between 2020 and 2023

**Fig. 1:**
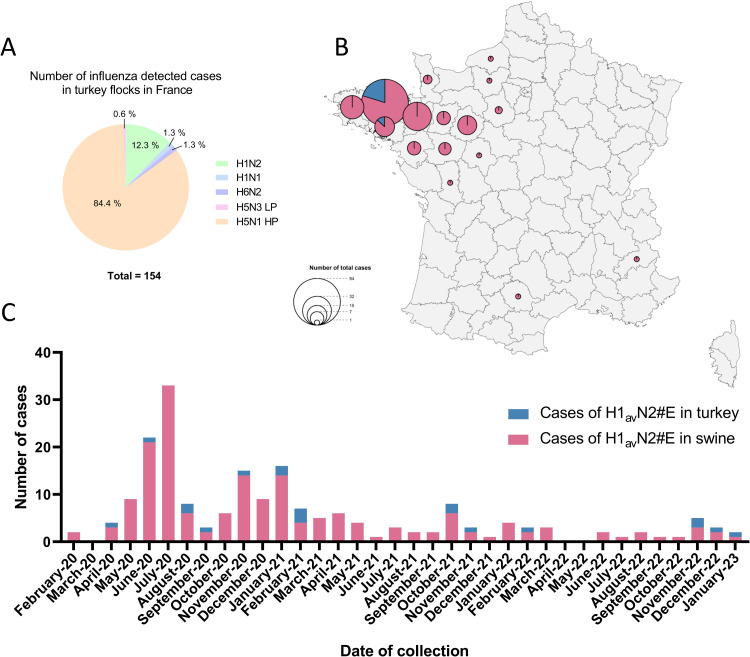
(A): Influenza subtypes detected in turkey flocks between February 2020 and January 2023 in France. (B): Spatial distribution and (C): temporal distribution of the influenza H1_av_N2#E cases in swine and turkey sequenced in France between February 2020 and January 2023. The pink color corresponds to cases in swine farms and the blue color corresponds to cases in turkey farms. The dates are the sampling dates of the swabs from the farms.

Between February 2020 and January 2023 in France, 154 positive influenza cases in turkey flocks were detected (Fig. 1A). Among these, 130 were confirmed as highly pathogenic H5N1 avian influenza cases. Other subtypes such as H5N3 and H6N2 low pathogenic avian influenza viruses were sporadically detected, as well as H1N1 from swine origin. Moreover, 19 flocks were tested positive for the H1avN2#E genotype. The 19 affected flocks were breeding turkeys located in the Brittany region exclusively, in two districts: “Côtes d’Armor” (89.5 % of the cases) in the north of the region and “Morbihan” (10.5 % of the cases) in the south of the region (Fig. 1B). The turkeys exhibited a marked drop in egg production, in some cases leading to a complete interruption of laying for several days, with no other associated clinical signs.

As previously described for swine cases, an epidemic phase occurred in 2020, followed by an enzootic phase beginning in 2021 (Richard, Herve, et al. 2025). Turkey cases were regularly detected during both phases (Fig. 1C) and were mostly located in the same location in Brittany (Fig. 1B). The two cases identified in the “Morbihan” district were detected at the end of the swine epizootic phase, in November 2020 and February 2021. In this district, 7.3 % of the total swine cases were reported. All other turkey cases were detected in the “Côtes-d’Armor” district which also accounted for the highest proportion of swine cases (37.9 % of total cases). During this period, NGS yielded the complete genome sequences for 19 turkey cases and 137 swine cases (Richard, Herve, et al. 2025).

**Fig. 2:**
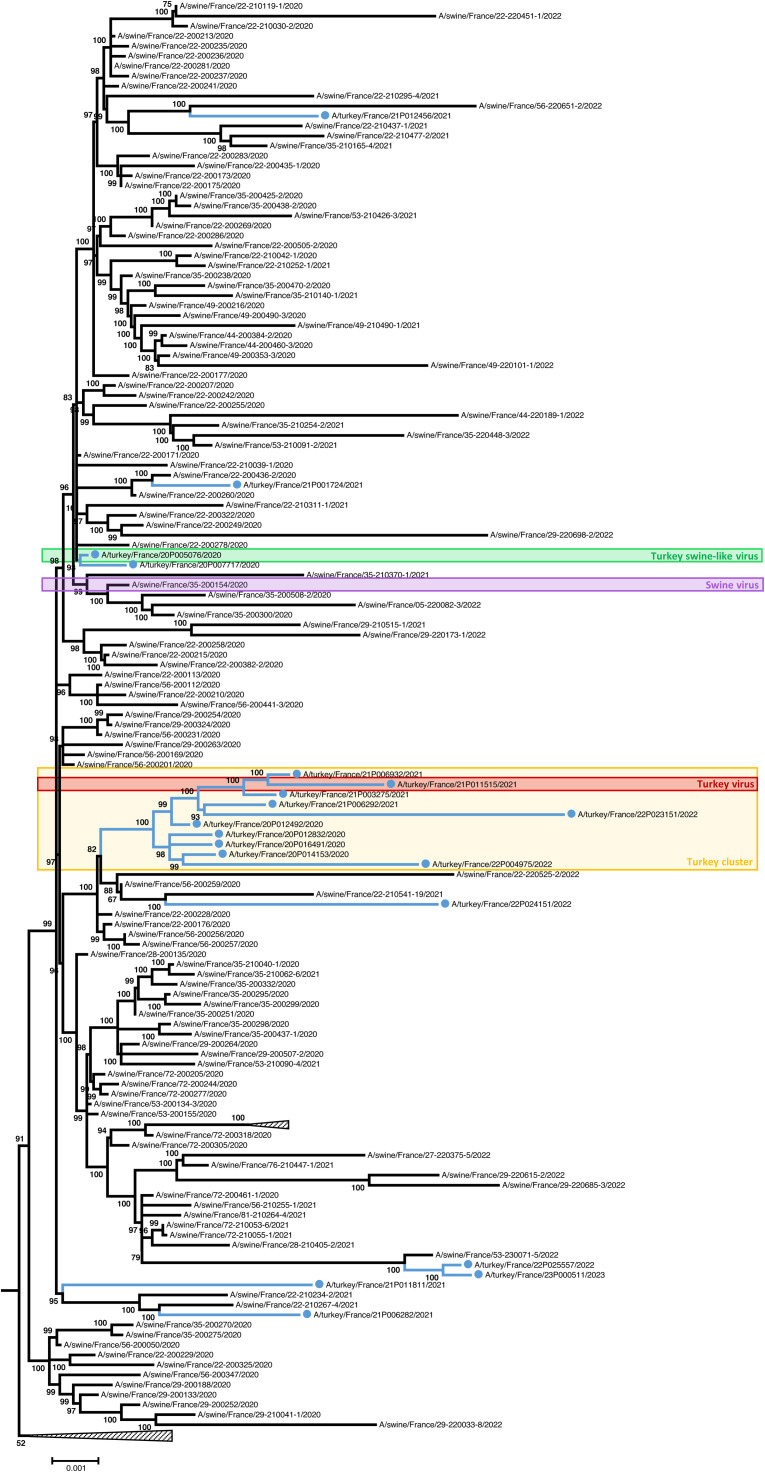
Maximum likelihood phylogeny of the 137 swine and 19 turkey influenza H1_av_N2#E complete genome sequences obtained from the 8 concatenated segments and detected between February 2020 and January 2023. The two H1_av_N2#E viruses from 2015 that root the tree are not shown. Blue lines with blue dots: turkey sequences. Yellow box: Turkey cluster. Green box: Turkey swine-like virus. Purple box: Swine virus. Red box: Turkey virus.

The phylogenetic tree (Fig. 2) showed that 9 out of the 19 sequences identified in turkeys between April 2020 and January 2023 in the “Côte d’Armor” district were sporadically detected among swine sequences and were phylogenetically very close to sequences detected in swine. This suggested occasional and isolated transmission events of the virus from swine to turkeys. The remaining 10 turkey sequences were grouped within a cluster composed exclusively of sequences detected in turkeys, supported by a bootstrap value of 100. These 10 sequences were detected between August 2020 and November 2022: 8 in the “Côtes-d’Armor” district and 2 in the “Morbihan” district. This "Turkey cluster" (yellow box) suggested that, following the cross-species transmission from swine to turkeys, the virus has subsequently circulated within turkey farms.

Two turkey strains have been isolated for this study, selected based on phylogenetic analyses. A reference swine strain A/swine/France/35–200,154/2020 previously selected by the swine influenza French NRL as a representative strain of swine H1_av_N2#E lineage was referred to as “Swine virus” (purple box) throughout the study (Deblanc et al. 2024). These viral strains are highlighted by colored boxes on the phylogenetic tree (Fig. 2). The turkey strain selected A/turkey/France/20P005076/2020 corresponded to the first detection of H1_av_N2#E in turkey. It was the first detected transmission from swine to turkey and thus appeared to be genetically close to these swine viral strains. As this virus was part of a group composed predominantly of swine sequences, it was referred to as “Turkey swine-like virus” (green box) throughout the study. The other turkey viral strain, A/turkey/France/21P011515/2021, was selected within the “Turkey cluster”, which was composed of sequences detected in turkeys exclusively. This viral strain was referred to as “Turkey virus” (red box) throughout the study. Based on its clustering with other turkey-derived sequences, it might have circulated within turkeys and might be adapted to this species.

### 3.2 Amino acid sequences analysis of influenza H1_av_N2#E circulating in France between 2020 and 2023

**Fig. 3:**
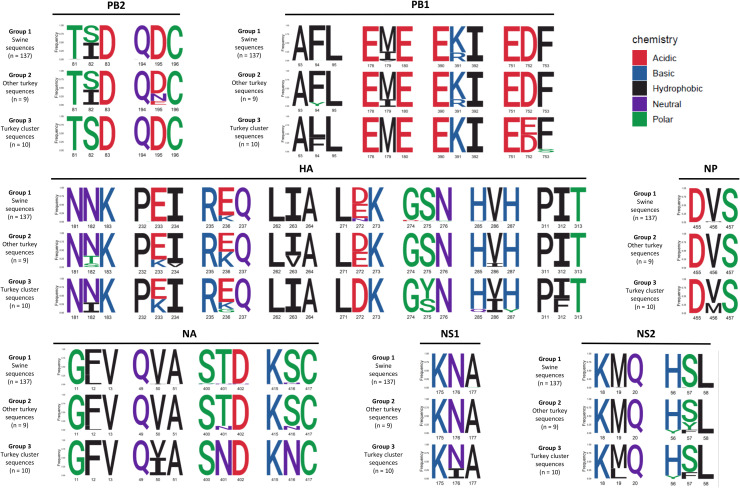
Comparison of amino acid frequency of the virus proteins for the three groups. All the positions on the protein (+/- 1 amino acid) for which the difference in proportion between at least two groups is greater than 25% are shown. Group 1: Swine sequences. Group 2: Other turkey sequences, outside the turkey cluster. Group 3: Turkey cluster sequences. The size of the letters is proportional to the occurrence of the amino acid at a given position.

The H1_av_N2#E influenza virus protein sequences from swine and turkeys were closely related. The maximum percentages of amino acid differences in the different proteins (according to the protein size) ranged from 1.4 % in the PB2 (Polymerase Basic 2) protein (total length: 759 amino acids) to 5.2 % in the PB1-F2 (Polymerase Basic 1 – F2) protein (total length: 58 amino acids). The highest number of amino acid differences between the strains was observed in the HA protein, with up to 22 substitutions (3.9 % of maximum amino acid differences, total length: 563 amino acids). Based on phylogenetic analysis and the host species, three groups were defined. Group 1 included all the 137 sequences detected in swine and was referred to as “Swine sequences”. Group 2 included the 9 turkey sequences detected among swine sequences and outside the turkey cluster and was referred to as “Other turkey sequences”. Group 3 included the 10 turkey sequences detected within the turkey cluster and was referred to as “Turkey cluster sequences”. Most of the amino-acid differences between the three groups were located in HA protein with 8 amino acid positions showing more than 25 % of difference: N182I, E233K, E236K, I263V, E272D, S275Y, V286I and I312F. On PB1 and NA proteins, 4 amino acid positions showed 25 % of difference (Fig. 3). No position showed more than 25 % of difference between at least two groups on the PB1-F2, PA (Polymerase Acid), PA-X, M1 (Matrix) and M2 proteins.

In the HA protein, positions 233 and 236 in the 220-loop of the RBS differed in frequencies in each group. Indeed, in group 1 (Swine sequences), at position 233, 99.3 % of the sequences presented a glutamate (E). This proportion decreased in group 2 (Other turkey sequences), with a glutamate (E) for 77.8 % and a lysine (K) for 22.2 %. In group 3 (Turkey cluster sequences), this proportion decreased further with a glutamate (E) for 60 % and a lysine (K) for 40 %. At position 236, 82.5 % of the sequences from group 1 presented a glutamate (E), 16.8 % a lysine (K) and 0.7 % a glycine (G). The proportion of glutamate (E) decreased in turkey sequences reaching 55.6 % in group 2 and 70 % in group 3. The frequency of lysine (K) increased in groups 2 and 3 compared to swine sequences with 44.4 % and 20 %, respectively. In group 3 sequences, 10 % exhibited a glycine (G) at this position.

Interestingly, when comparing all turkey sequences to each other from both groups, 63.2 % showed one mutation on positions 233 or 236, from a glutamate (E) to a lysine (K). This phenomenon was observed in only 23.2 % of the sequences detected in swine. Two positions in the HA protein exhibited amino acid frequency differences greater than 50 % between at least two groups. At position 272, 48.2 % of group 1 sequences had an aspartate (D), 42.3 % had a glutamate (E) and 9.5 % had an asparagine (N). In group 2 sequences, 55.6 % had an aspartate (D) and 44.4 % had a glutamate (E). In group 3 sequences, 100 % had an aspartate (D). At position 275, 97.8 % of group 1 and 100 % of group 2 sequences had a serine (S), in contrast with sequences of group 3 which had 40 % of serine (S) and 60 % of tyrosine (Y).

In NA protein, the two positions 401 and 416 showed a nearly complete amino acid change between the sequences of groups 1 and 2 and the sequences of group 3. At position 401, 97.8 % of group 1 sequences had a threonine (T), while only 2.2 % had an asparagine (N). The proportion of threonine (T) decreased slightly in group 2 to 88.9 %, and 11.1 % had an asparagine (N). In group 3, 100 % of the sequences presented an asparagine (N). This position 401 is located in the NA antigenic site 2d At position 416, with nearly same frequencies as position 401, 94.9 % of group 1 sequences presented a serine (S), while only 4.4 % presented an asparagine (N). The proportion of serine (S) slightly decreased in group 2 to 88.9 %, and 11.1 % showed an asparagine (N). In group 3, 100 % of the sequences presented an asparagine (N).

The *in silico* analysis of glycosylation sites revealed five potential N-glycosylation sites on HA and five on NA. Most of these sites were conserved across the three groups. However, in the HA protein, position 286 (substituted from valine (V) to isoleucine (I) in 40 % of the group 3 was located immediately upstream of the glycosylation site at position 288. No other positions of interest or antigenic sites overlapped with predicted glycosylation motifs.

In PB1 protein, position 94 exhibited amino acid frequency differences greater than 50 % between at least two groups. In group 1 sequences, 99.3 % presented a phenylalanine (F). In group 2 sequences, 88.9 % had a phenylalanine (F), and 11.1 % a tyrosine (Y). Only 40 % of group 3 sequences had a phenylalanine (F), and 60 % a leucine (L).

Regarding the antigenic sites described on HA and NA, except in the 220-loop of the RBS on HA and at position 401 on NA as mentioned above in this section, no major differences were observed between the two groups (Supplementary Fig. 9).

All amino acid positions showing differences between the three sequence groups were summarized together with their relative frequencies, the protein domains in which they are located, the reported functions of these domains and relevant references (Table 1).

**Table 1:** Summary of amino acid positions showing differences between the three sequence groups, their frequencies within each group and the corresponding protein domains with known domain functions.

Protein	Position	Group 1 Swine sequences (AA %) n = 137	Group 2 Other turkey sequences (AA %) n = 9	Group 3 Turkey cluster sequences (AA %) n = 10	Note about this position	Reference
PB2	82	I (53.3 %), S (46 %)	S (55.6 %), I (44.4 %)	S (100 %)	N-terminal domain (N1 linker domain) Binding domain with C-terminal region of PB1 and thumb domains Guiding of the vRNA 3′ terminus into active site	(Szeto et al. 2020) (Wang et al. 2016) (Pflug et al. 2014)
	195	D (100 %)	D (66.7 %), N (22.2 %), E (11.1 %)	D (100 %)	N-terminal domain (Lid domain) Stabilizating structure	
PB1	94	F (99.3 %)	F (88.9 %), Y (11.1 %)	L (60 %), F (40 %)	Fingers domain Interaction domain with PA protein	(Pflug et al. 2014) (Carter and Iqbal 2024)
	179	M (71.5 %), I (25.5 %)	M (77.8 %), I (22.2 %)	M (100 %)	At the junction between fingers and beta-ribbon domains Interaction domain with PA, contains Nuclear Localization Signals (NLSs)	
	391	K (74.5 %), R (25.5 %)	K (77.8 %), R (22.2 %)	K (100 %)	Beta-hairpin domain Binding cleft for the vRNA 5′ terminus	
	752	D (100 %)	D (100 %)	D (50 %), E (50 %)	C-terminal domain Binding domain with N-terminal region of PB2 (N-domain) and with vRNA 3′ terminus	
HA	182	N (99.3 %)	N (66.7 %), T (22.2 %), S (11.1 %)	N (70 %), I (30 %)	HA1 subunit: Antigenic site - *Ca* 1 Target for neutralizing antibodies	(Sriwilaijaroen and Suzuki 2012) (N.C. Wu et al. 2017) (Y. Sun et al. 2021)
	233	E (99.3 %)	E (77.8 %), K (22.2 %)	E (60 %), K (40 %)	HA1 subunit: RBS - 220-loop HA binding to sialic acid receptors	
	236	E (82.5 %), K (16.8 %)	E (55.6 %), K (44.4 %)	E (70 %), K (20 %), G (10 %)	HA1 subunit: RBS - 220-loop HA binding to sialic acid receptors	
	272	D (46.7 %), E (43.8 %), N (9.5 %)	D (55.6 %), E (44.4 %)	D (100 %)	HA1 subunit: Upstream of the vestigial esterase domain Function not well defined Structural stabilization of HA head	(Zheng et al. 2018) (Ghafoori et al. 2023)
	275	S (97.8 %)	S (100 %)	Y (60 %), S (40 %)	HA1 subunit: Near the vestigial esterase domain Function not well defined Target for antibodies	
	286	V (98.5 %)	V (88.9 %)	V (60 %), I (40 %)	HA1 subunit: Inside the vestigial esterase domain Function not well defined Target for antibodies	
	312	I (100 %)	I (100 %)	I (60 %), F (40 %)	HA1 subunit: Fusion F' subdomain, upstream of the cleavage site Stabilization of HA0, membrane fusion	(Sriwilaijaroen and Suzuki 2012) (Carter and Iqbal 2024)
NP	456	V (97.8 %)	V (100 %)	V (70 %), M (30 %)	C-terminal domain Tail loop and helix-loop-helix motif for trimerization	(Ng, Wang, and Shaw 2009)
NA	12	F (72.3 %), L (27 %)	F (88.9 %), L (11.1 %)	F (100 %)	N-terminal domain: transmembrane domain Signals for translocation from the endoplasmic reticulum to the surface and association with lipid rafts	(da Silva et al. 2013) (Webster, Brown, and Laver 1984) (McAuley et al. 2019)
	50	V (97.1 %)	V (100 %)	V (50 %), I (50 %)	N-terminal domain: stalk, near disulfide bonds Tetramer stabilization	
	401	T (97.8 %)	T (88.9 %), N (11.1 %)	N (100 %)	Head domain: antigenic site 2d Adjacent to catalytic pocket and target for antibodies	
	416	S (94.9 %)	S (88.9 %), N (11.1 %)	N (100 %)	Head domain Adjacent to catalytic pocket and target for antibodies	
NS1	176	N (100 %)	N (100 %)	N (70 %), I (30 %)	Effector domain: CPSF30 binding pocket Virulence determinant by blocking 3′ processing of cellular mRNAs Stabilization of the effector domain	(Noah, Twu, and Krug 2003) (Twu et al. 2006) (Kim, Jeong, and Jang 2021)
NS2	19	M (98.5 %)	M (100 %)	M (70 %), L (30 %)	N-terminal domain: Nuclear Export Signal (NES) region Role in RNP nuclear export	(Neumann, Hughes, and Kawaoka 2000) (Akarsu et al. 2003) (Paterson and Fodor 2012)
	57	S (98.5 %)	S (66.7 %), Y (22.2 %), F (11.1 %)	S (80 %), F (20 %)	Beginning of C-terminal domain Interaction with M1 protein	

### 3.3 Selection pressure analysis of influenza H1avN2#E circulating in France between 2020 and 2023

**Fig. 4:**
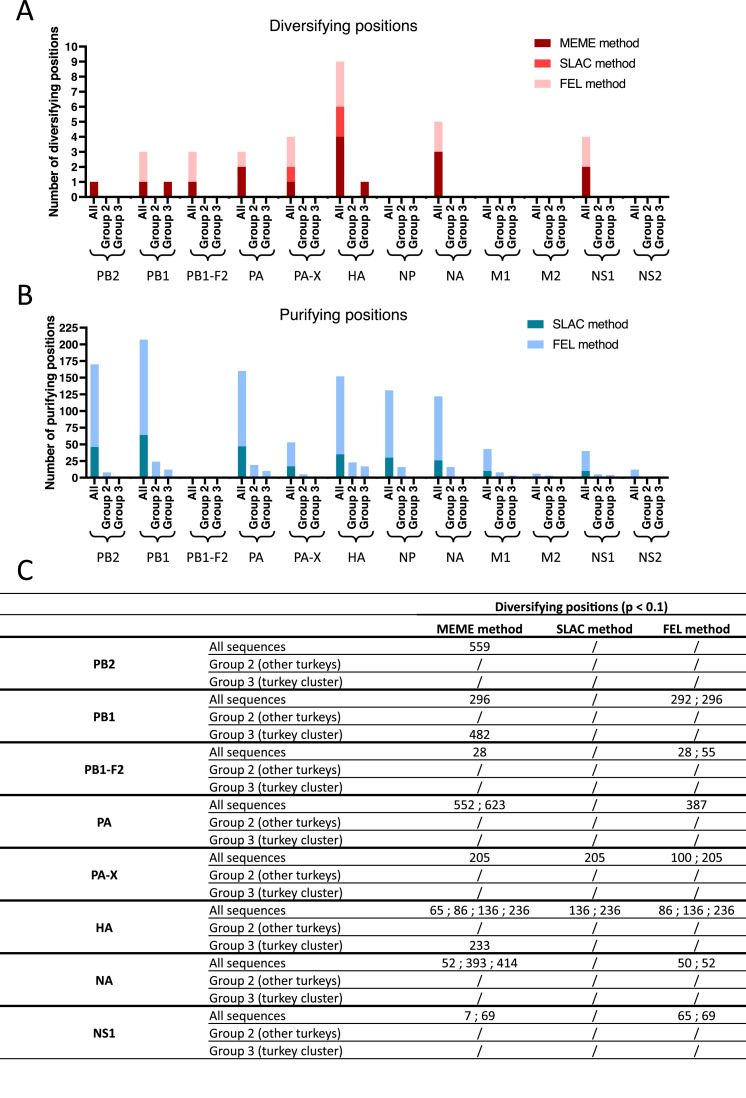
Selection pressure analyses on the sites of the 12 major proteins for all sequences ("All"), sequences detected in turkeys outside the turkey cluster ("Group 2"), and sequences detected in turkeys within the turkey cluster ("Group 3"). (A) Diversifying sites, identified using MEME (dark red), SLAC (medium red), and FEL (light red). (B) Purifying sites, identified using SLAC (dark blue) and FEL (light blue). (C) Table showing the detailed diversifying sites for the same subsets and the same three methods on the 8 proteins that displayed at least one diversifying site.

Purifying and diversifying sites for the 12 major proteins were investigated for the following three subsets: all sequences, Group 2 sequences (turkey sequences outside the turkey cluster) and Group 3 sequences (turkey sequences within the turkey cluster) (Fig. 4). The choice of these analysis groups was based on the potential adaptation of mutations to the turkey host. Across all proteins and the three subsets, selection was dominated by strong purifying pressure. Using the SLAC method, we detected 293 purifying sites (p(dN < dS) < 0.1) and 3 diversifying sites (p(dN > dS) < 0.1) across all proteins and subsets combined (Figs. 4A and 4B). The FEL method classified 984 sites as purifying (β < α, p < 0.1) and 14 sites as diversifying (β > α, p < 0.1). For both methods, the majority of diversifying positions were located in HA. Episodic positive selection signals (MEME method) amounted to 17 across all proteins and subsets (p < 0.1) with the majority also located in HA (Fig. 4A).

Within HA, the MEME method identified four positions under episodic selection across all sequences, at positions 65, 86, 136 and 236 (Fig. 4C). Positions 136 and 236 were also identified with SLAC and FEL methods and position 86 with FEL. Interestingly, position 233 was identified as a diversifying site only in the turkey cluster sequences (Group 3). This position, located within the 220-loop of the RBS, was also identified as of interest in terms of amino acid frequencies in the previous analysis. The only other diversifying position identified by MEME in the turkey cluster sequences was position 482 in PB1, which was not confirmed by the amino acid frequency analysis. In the turkey sequences outside the turkey cluster (Group 2), no diversifying positions were detected with any of the three methods.

### 3.4 Nucleotide and amino acid analyses of the three studied viruses for *in vitro* assays

The three studied viruses each originated from one of the three groups mentioned above. The reference swine virus, “Swine virus”, originated from group 1 (Swine sequences). The two viruses detected in turkeys, the “Turkey swine-like virus” and the “Turkey virus”, originated from groups 2 (Other turkey sequences) and 3 (Turkey cluster sequences), respectively. A comparison of the nucleotides and amino acid sequences for the virological characterization was performed on these three selected strains.

The “Turkey swine-like virus” and the “Swine virus” were very similar and differed by 14 nucleotides on 13,588 across the entire genome. The complete protein sequences of these two viruses shared 99.96 % of identity. Only two nucleotide mutations altered the amino acid between them: R189K on PB1 gene and K233E on HA gene. The “Turkey virus” showed more differences from the first two viruses. It exhibited 99.23 % of identity with the “Swine virus”, which was the most divergent virus. It had 34 and 35 amino acid differences on 4789 across the entire proteome with the “Turkey swine-like virus” and the “Swine virus”, respectively. More specifically, there were 8 differences in the HA protein, 5 and 4 in the PB1 protein with the “Swine virus” and “Turkey swine-like virus” respectively, 5 in the NA protein, 4 in the NS1 (Non-Structural 1) protein, 3 in the NS2 (Non-Structural 2) protein, 3 in the PB2 protein, 3 in the NP (Nucleoprotein) protein, 2 in the PB1-F2 protein, one on the PA protein, one in the PA-X protein, and none in the M protein. Among these differences, 68 % corresponded to the mutations of interest described in Fig. 3.

### 3.5 Comparison of sequences from field samples and after viral production

Sequences of the viral stocks produced for *in vitro* studies were compared to those obtained from the field samples. After viral replication of the turkey and swine viruses on MDCK cells, the “Turkey virus” exhibited two non-synonymous mutations in the HA gene compared to initial sequence obtained from field sample. These two mutations were K233M and E236G. Interestingly, these two mutations were located in the antigenic site of the 220-loop in the RBS, at the same positions where amino acid differences were observed between group 1 (Swine sequences), group 2 (Other turkey sequences) and group 3 (Turkey cluster sequences). The “Swine virus” and the “Turkey swine-like virus” did not exhibit any mutations resulting in amino acid changes after viral replication on MDCK cells.

### 3.6 Viral genomic loads and infectious titers during replication kinetics on cells

**Fig. 5:**
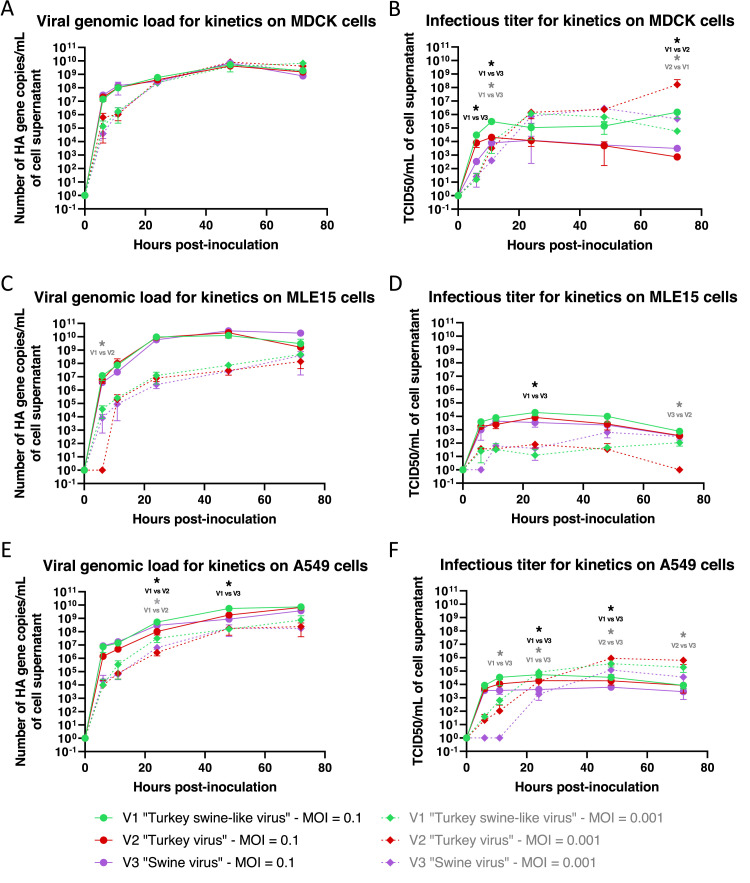
Evolution of the viral genomic load (left panel) and the viral infectious titer (right panel) on cells over time post-infection. Comparison of the three viruses on MDCK cells (A and B), MLE15 cells (C and D) and A549 cells (E and F). Solid lines correspond to MOI 0.1 and dashed lines to MOI 0.001. *: p < 0.05, Kruskal–Wallis test. Black asterisk: significant difference at MOI 0.1; grey asterisk: significant difference at MOI 0.001.

On MDCK cells, at the same MOI, no significant differences were observed for the viral genomic load between the three viruses (Fig. 5A). Moreover, similar levels were observed at MOIs of 0.1 and 0.001 after 24 hpi. The genomic replication reached a plateau at 48 hpi with approximately 10^9^ HA gene copies per mL of cell supernatant. At a MOI of 0.1, the infectious titers (Fig. 5B) of the “Turkey swine-like virus” seemed to reach a plateau at 11 hpi, earlier than the viral genomic load, with a titer of 10^5^ TCID_50_/mL of cell supernatant. At MOI 0.1, the infectious titers of the “Turkey swine-like virus” was significantly higher compared to the “Swine virus” and the “Turkey virus” which reached a lower plateau at 11 hpi with around 10^4^ TCID_50_/mL, and then declined to 10^3^ TCID_50_/mL at 72 hpi. At MOI 0.001, the plateau was reached later, at 24 hpi for all three viruses. The infectious titers were higher than at MOI 0.1 with around 10^6^ TCID_50_/mL of supernatant for the three viruses between 24 and 48 hpi. At 72 hpi, the “Turkey virus” reached a peak of 10^8^ TCID_50_/mL, statistically higher than the “Turkey swine-like virus”. In MDCK cells, despite similar viral genomic loads for both MOIs, infectious titers were higher at MOI 0.001 than at MOI 0.1 from 24 hpi for all three viruses.

In contrast to what observed on MDCK cells, viral genomic loads on MLE15 cells showed distinct genomic replication kinetics depending on the MOI. At MOI 0.1, viruses rapidly reached a plateau at 24 hpi near 10^10^ HA gene copies/mL, while at MOI 0.001, there was a delay and the plateau was reached later at 72 hpi, around 10^8^ HA gene copies/mL (Fig. 5C). Concerning infectious titers (Fig. 5D), at MOI 0.1, the plateau was reached at 24 hpi with 10^3.5^ TCID50/mL, where the infectious titer of the “Turkey swine-like virus” was statistically higher than that of the “Swine virus”. Furthermore, the “Turkey swine-like virus” showed a slightly higher titer throughout the kinetic, but without significant differences at other time points. At MOI 0.001, the infectious titers were lower for all the three viruses compared to MOI 0.1 and to what observed on MDCK cells for the same MOI. In MLE15 cells, as observed for viral genomic loads, infectious titers remained consistently higher at MOI 0.1 than at MOI 0.001 throughout the kinetics for all three viruses.

In A549 cells, viral genomic loads also showed distinct genomic replication kinetics depending on the MOI. At MOI 0.1, viruses reached lately a plateau at 72 hpi near 10^9^ HA gene copies/mL while at MOI 0.001, there was a delay and the plateau was also reached at 72 hpi, near 10^8^ HA gene copies/mL (Fig. 5E). A difference was observed between the viruses: the “Turkey swine-like virus” showed a higher genomic load than the “Turkey virus” at 24 hpi and than the “Swine virus” at 48 hpi. At MOI 0.1, the infectious titer in A549 cells (Fig. 5F) quickly reached a plateau for the three viruses, around 11 hpi, and then infectious titers remained stable until 72 hpi. The titer was statistically higher at 24 and 48 hpi for the “Turkey swine-like virus” compared to the “Swine virus”. At MOI 0.001, the plateau was reached later, around 48 hpi, with a statistically higher titer for the “Turkey virus” compared to the “Swine virus”. In A549 cells, although viral genomic loads were higher at MOI 0.1 than at MOI 0.001 for all three viruses, infectious titers were higher at MOI 0.001 from 48 hpi.

### 3.7 Viral genomic loads and infectious titers during replication kinetics on embryonated chicken eggs

**Fig. 6:**
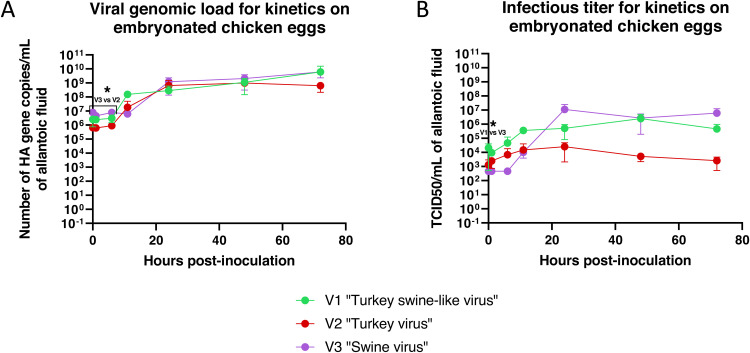
Evolution of the viral genomic load (A) and the viral infectious titer (B) on embryonated chicken eggs over time post-infection. *: p < 0.05, Kruskal–Wallis test.

In embryonated chicken eggs, the viral genomic load reached a plateau at 24 hpi for the “Turkey virus” at approximately 10^9^ HA gene copies/mL, which corresponded to an increase of 3 log_10_ of the load present at 0 hpi. For the “Turkey swine-like virus” and the “Swine virus”, the plateau was reached later, at 72 hpi with approximately 10^9.5^ HA gene copies/mL, about 10^3^ and 10^2.5^ times higher than the loads observed at 0 hpi, respectively (Fig. 6A). No significant differences were observed between the three viruses from 11 hpi onwards. The viral infectious titers displayed a different profile (Fig. 6B). The “Turkey virus” showed very low production of infectious particles, with only a 10-fold increase at 24 hpi compared to 0 hpi, followed by a decline in production. The “Turkey-swine-like virus” reached a plateau at 24 hpi, at approximately 10^6.5^ TCID_50_/mL, corresponding to about a 10^2.5^-fold increase compared to 0 hpi. Finally, the “Swine virus” reached a plateau as early as 24 hpi, at 10^7^ TCID_50_/mL, more than 10^4^ times higher than the load present at 0 hpi. Again, no significant differences were observed between the three viruses.

## 4 Discussion

Since 2020, the influenza H1avN2#E virus has become the predominant strain in French pig farms (Richard, Herve, et al. 2025), with sporadic detections in 19 breeding turkey flocks. From summer 2021, it appeared endemic in swine. In addition to the reported human case, phylogenetic and epidemiological data suggested recurrent swine-to-turkey transmission events. Within One Health context, monitoring viruses with zoonotic potential and species barrier crossings, as demonstrated here, is essential. Although less frequent than swine-to-swine spread, spillover of IAVs to poultry, particularly turkeys, is not anecdotal. In 2020, Brittany alone hosted more than 4000 pig farms and nearly 600 turkey farms, representing 56 % and 7 % of the national production respectively (Agreste 2024a, 2024b). Their geographic proximity likely increases indirect contacts and thus interspecies transmission risk.

Several hypotheses can be proposed regarding transmission routes between swine and turkey flocks. The main excretion pathway of IAVs in swine is respiratory via oral and nasal secretions, particularly through infected microdroplets, with minimal shedding in feces (Brown 2000). In infected swine herds, infectious virus has been isolated only from air samples collected inside barns or at exhaust fans, while viral RNA can be detected further downwind up to 2 km (Corzo et al. 2013). Experimental studies further confirm that airborne transmission of IAV in pigs is limited to very short distances (Zhang et al. 2013). In Brittany, where swine and turkey farms are densely distributed, the transport of shedding swine in open trucks near turkey farms may also facilitate transmission. Movement of personnel, animals or equipment can contribute to viral spread, although direct epidemiological links between swine and turkey farms in France remain limited. An *in vivo* study using an H1_av_N2#E strain showed no enteric tropism in swine (Deblanc et al. 2024) and an Anses (scientific expertise agency that monitors and assesses health risks) report considered persistence of infectious virus in stored swine slurry unlikely (Anses 2024). Nevertheless, slurry may become contaminated with respiratory secretions, enabling viral particles to survive during spreading. Supporting this, IAVs persisted up to seven weeks in duck slurry (Schmitz et al. 2020) and under cold storage conditions, particularly in winter, persistence cannot be excluded (Tun, Cai, and Khafipour 2016; Li and Robertson 2021). Field epidemiological investigations are therefore warranted, including testing of slurry, surface swabs and air samples from infected swine and turkey farms, combined with molecular epidemiology and movement data to identify potential transmission links.

Phylogenetic analyses showed that 9 of the 19 turkey sequences were sporadically detected among swine sequences, consistent with 9 independent swine-to-turkey transmission events of the H1avN2#E virus. In contrast, 10 turkey sequences clustered together without swine sequences suggested circulation between turkey farms. Similar inter-farm IAV transmission among turkey flocks has been reported elsewhere (Mathieu et al. 2021; Mehrabadi et al. 2018), likely due to biosecurity breaches or infected peridomestic animals.

The presence of both α2,3- and α2,6-linked SA receptors in the turkey respiratory epithelium, similar to swine, may facilitate attachment of swine-origin strains and interspecies transmission. This receptor distribution likely explains the ability of swine influenza viruses to spread to turkeys, but not to chickens, despite the high density of chicken farms. Age-related increases in α2,6-linked SA receptors in turkeys may also account for detection of this virus mainly in breeding rather than fattening flocks (Pillai and Lee 2010; Kimble, Nieto, and Perez 2010). Nevertheless, circulation in fattening flocks cannot be excluded, even though annual serological surveys have reported few positive cases in these flocks. The absence of clear clinical signs, other than reduced egg production, which does not apply to fattening flocks, may have allowed unnoticed viral spread.

Comparison of viral protein sequences from swine, non-cluster turkey and turkey cluster revealed several differences, notably in HA and NA. Mutations E233K and E236K were particularly relevant as they were located in the 220-loop of the HA RBS, a domain critical for SA binding and viral fitness (Wu et al. 2017). Although these positions have not been specifically described in the literature, they may be under selective pressure. Positive selection analyses further supported residue 233 which was diversifying only within the turkey cluster, consistent with a potential E233K adaptation to turkeys. In contrast, residue 236 was diversifying across all sequences but not specific to turkeys. At least one glutamate-to-lysine change at positions 233 or 236 characterized two-thirds of the turkey sequences but less than one-quarter of the swine sequences, suggesting adaptation feature. This substitution alters amino acid properties, replacing an acidic glutamate with a basic lysine, potentially affecting HA conformation and function. Such changes can significantly influence infectivity (Cotter, Jin, and Chen 2014).

On NA protein, mutations T401N and S416N were of particular interest, especially T401N within antigenic site 2d, adjacent to the catalytic pocket and targeted by neuraminidase-inhibiting antibodies (Webster, Brown, and Laver 1984). These substitutions represented near-complete shifts between the two turkey groups and, by altering polarity and steric hindrance, may affect NA conformation, antigenicity, immune escape and enzymatic activity. Despite this, positive selection analyses did not detect diversifying pressure at these sites, suggesting that they may have become fixed through drift or host-specific constraints, while still potentially influencing protein structure, enzymatic function and immune recognition. Additional mutations with more than 50 % frequency differences between groups included E272D and S275Y in HA and F92L in PB1. Although not located in antigenic sites, the HA changes could alter protein conformation and indirectly affect receptor binding. However, as with NA residues 401 and 416, no evidence of diversifying selection was detected.

Using MEME, FEL and SLAC methods allowed complementary perspectives: MEME detected episodic events while FEL and SLAC highlighted pervasive or purifying effects. Across the 12 proteins, analyses consistently indicated dominant purifying selection with few signals of diversification. The only evidence of potential adaptation to turkeys was position 233 in HA, also highlighted by amino acid frequency analyses. The absence of selection elsewhere, including in NA despite near-complete shifts at residues 401 and 416, suggested fixation by drift rather than adaptive pressure. Together, these approaches confirmed the predominance of purifying selection with isolated rare diversifying episodes compatible with host-specific adaptation in turkeys.

Glycosylation in HA and NA can modulate antibody sensitivity, stability, and virulence (Sun et al. 2013). The V286I mutation on HA, although not abolishing the downstream consensus motif, could affect glycosylation efficiency or accessibility, and thus contribute to adaptation.

The three mammalian cell lines used for viral and genomic replication kinetics (MDCK, MLE15 and A549) all supported efficient replication of the studied H1avN2#E viruses. This confirms their relevance for investigating viruses crossing the species barrier. Notably, replication was robust in human lung cells, consistent with the documented human case and in murine lung cells, supporting their use as an accessible infection model. MDCK cells are well established for influenza virus studies (Lombardo et al. 2012) while A549 cells provide a robust system to study swine influenza (Cheng et al. 2022), human influenza (Zhuravlev et al. 2022) and avian influenza viruses (Yang et al. 2021). Replication was evaluated at both low (0.001) and higher (0.1) MOIs. Although all cell types supported virus growth, genomic loads and infectious titers differed. These discrepancies reflected the different and necessary TPCK-trypsin concentrations used (2, 1 and 0.2 µg/µL for MDCK, MLE15 and A549 respectively) to avoid cytotoxicity. Trypsin cleavage of HA0 into HA1 and HA2 subunits is essential for viral entry (Klenk et al. 1975; Steinhauer 1999) and higher concentrations facilitate more particle release.

Viral and genomic replication kinetics in embryonated chicken eggs showed that this model supported replication but at much lower efficiency than mammalian cell lines. Infectious titers increased at most 10^4^-fold for the “Swine virus” compared with up to 10^8^-fold in cells while genomic load rose only 10^3^-fold. Considerable variability was observed between replicates, likely because each egg corresponded to a single embryo unlike homogeneous cell cultures where numerous cells ensured reproducibility. Using a larger number of eggs per condition would probably increase the statistical power. Despite no statistically significant difference, the “Turkey virus” led to fewer infectious particles than the two other viruses. Although phylogenetic analyses suggested adaptation to turkeys, embryonated chicken eggs did not allow efficient replication. The “Turkey virus” differed from the “Turkey-swine-like virus” and the “Swine virus” by 34 and 35 amino acids out of 4789 respectively, mainly in HA (8 substitutions), NA (5 substitutions) and PB1 (4 and 5 substitutions respectively), any of which could contribute to reduced replication. Interestingly, after a single passage in MDCK cells, HA mutations K233M and E236G emerged in the “Turkey virus”, with residue 233 appearing under selective pressure in our analyses. These findings were consistent with a readaptation process from the avian host back to mammalian cells. This might explain the poor growth in eggs but higher production in mammalian cells, especially at low MOI.

These viral replication kinetics demonstrated that both MDCK and A549 cells supported efficient production of infectious particles at low MOI (0.001). The subtle differences observed between the three viruses can likely be attributed to their high degree of genetic similarity. Nonetheless, discrimination was possible: at low MOI, the “Turkey virus” produced higher titers, while at high MOI, the “Turkey-swine-like virus” yielded more infectious particles. In embryonated eggs, the “Turkey-swine-like virus” and “Swine virus” produced higher levels of infectious particles than the “Turkey virus”. Although these swine-origin viruses were not tested on porcine cells here, collaborators observed minimal replication in NPTr cells (data not shown) and T3 porcine respiratory epithelial cells may represent a more suitable model (Avanthay et al. 2023).

The discrepancy between genomic loads and infectious titers likely reflects several factors. Viral RNA from degraded particles can persist and be detected by rRT-qPCR and not all particles produced are infectious. Defective interfering particles (DIPs), well described in negative-strand RNA viruses including IAVs (M. Wu et al. 2022), may account for this, particularly in embryonated eggs and at high MOI in cell cultures, where infectious titers were consistently more than 1000-fold lower than genomic loads.

To further characterize these viruses, more complex models should be considered. *In vitro* respiratory organoids can reproduce the diversity and interactions of respiratory cells (Zhou et al. 2018) while *in vivo* models take into account the complete interactions that actually occur in the organism and better reflect farm conditions. Characterizing the two turkey viruses in breeding turkeys, where they naturally occur, would be highly relevant. The “Swine virus” has already been studied in pigs (Deblanc et al. 2024), but testing the turkey viruses in swine would also be valuable, as spillback from turkeys cannot be excluded. The murine model, although not a natural host for IAVs, remains a practical and low-cost model with well-documented susceptibility to IAVs (Shope 1935; Driskell et al. 2010), likely due to a respiratory receptor distribution favorable to avian strains (Ibricevic et al. 2006). In a context of crossing the species barrier of these swine-origin viruses, then transmitted to turkeys and to other mammals, the mouse would therefore be an interesting study model.

In conclusion, this study demonstrated swine-to-turkey spillovers of the H1avN2#E virus, first identified in swine in 2020, and provided evidence of possible turkey-to-turkey transmission. A glutamate-to-lysine substitution at positions 233 or 236 of the HA RBS 220-loop may represent an adaptation marker for turkeys. Similarly, NA substitutions T401N in antigenic site 2d and S416N may represent a feature associated with turkeys, despite the absence of detectable positive selection. Viral and genomic replication kinetics revealed subtle differences among the three viruses, with the “Turkey virus” producing higher titers in MDCK and A549 cells at MOI 0.001 while the “Turkey-swine-like virus” yielded more at MOI 0.1. In embryonated chicken eggs, infectious particles were produced for the “Turkey-swine-like virus” and “Swine virus” but not efficiently for the “Turkey virus”. Further characterization of these H1N2 viruses in additional host-derived cells and *in vivo* models would be needed to better capture the complexity of host-virus interactions.

## Funding

This work was funded by Anses (French Agency for Food, Environmental and Occupational Health & Safety) and INRAE (French National Research Institute for Agriculture, Food and Environment).

## CRediT authorship contribution statement

**Chloé Chavoix:** Writing – review & editing, Validation, Supervision, Resources, Project administration, Methodology, Investigation, Formal analysis, Conceptualization. **Pascale Massin:** Writing – review & editing, Validation, Supervision, Resources, Project administration, Methodology, Investigation, Formal analysis, Conceptualization. **François-Xavier Briand:** Writing – review & editing, Visualization, Validation, Software, Resources, Methodology, Formal analysis, Data curation, Conceptualization. **Katell Louboutin:** Investigation. **Rachel Busson:** Investigation. **Florent Souchaud:** Investigation. **Gautier Richard:** Writing – review & editing, Resources, Data curation. **Claire Martenot:** Writing – review & editing, Resources. **Aurélie Le Roux:** Investigation. **Edouard Hirchaud:** Investigation. **Yannick Blanchard:** Writing – review & editing, Resources. **Sophie Le Bouquin-Leneveu:** Resources. **Axelle Scoizec:** Resources. **Céline Deblanc:** Writing – review & editing, Resources. **Séverine Hervé:** Writing – review & editing, Resources. **Audrey Schmitz:** Writing – review & editing, Resources. **Eric Niqueux:** Writing – review & editing, Resources. **Gaëlle Simon:** Writing – review & editing, Resources, Conceptualization. **Ronan Le Goffic:** Writing – review & editing, Validation, Supervision, Project administration, Methodology, Funding acquisition, Conceptualization. **Béatrice Grasland:** Writing – review & editing, Validation, Supervision, Resources, Project administration, Methodology, Funding acquisition, Conceptualization.

## Declaration of competing interest

The authors declare that they have no known competing financial interests or personal relationships that could have appeared to influence the work reported in this paper.

The authors declare the following financial interests/personal relationships which may be considered as potential competing interests:

## Data Availability

All turkey sequences have been deposited to GISAID database and accession numbers are available in Supplementary Table 1. Other data will be made available on request.

## Appendix Supplementary materials

Image, application 1
