# Supplementary material for: Identification of H1N2 influenza viruses in turkeys after spillover from swine and in vitro characterization
Source: Virus Res. 2025 Sep 21;361:199634. doi: 10.1016/j.virusres.2025.199634 (PMC12506539; doi:10.1016/j.virusres.2025.199634)
Supplement: Supplementary file 1 [file mmc1.zip › Supplementary_Table_1.docx]

**Supplementary Table 1.** GISAID accession numbers for the 19 H1_av_N2#E sequences isolated in turkey in France from April 2020 to January 2023 and used in this study.

| Strain name | Collection date | Isolate Id | PB2 segment | PB1 segment | PA segment | HA segment | NP segment | NA segment | M segment | NS segment |
| --- | --- | --- | --- | --- | --- | --- | --- | --- | --- | --- |
| A/turkey/France/23P000511/2023 | 2023-01-09 | EPI_ISL_20078624 | EPI4544080 | EPI4544081 | EPI4544079 | EPI4544083 | EPI4544076 | EPI4544082 | EPI4544078 | EPI4544077 |
| A/turkey/France/22P025557/2022 | 2022-12-09 | EPI_ISL_20078623 | EPI4544072 | EPI4544073 | EPI4544071 | EPI4544075 | EPI4544068 | EPI4544074 | EPI4544070 | EPI4544069 |
| A/turkey/France/22P024151/2022 | 2022-11-28 | EPI_ISL_20078622 | EPI4544064 | EPI4544065 | EPI4544063 | EPI4544067 | EPI4544060 | EPI4544066 | EPI4544062 | EPI4544061 |
| A/turkey/France/22P023151/2022 | 2022-11-03 | EPI_ISL_20078621 | EPI4544056 | EPI4544057 | EPI4544055 | EPI4544059 | EPI4544052 | EPI4544058 | EPI4544054 | EPI4544053 |
| A/turkey/France/22P004975/2022 | 2022-02-02 | EPI_ISL_20078620 | EPI4544048 | EPI4544049 | EPI4544047 | EPI4544051 | EPI4544044 | EPI4544050 | EPI4544046 | EPI4544045 |
| A/turkey/France/21P012456/2021 | 2021-11-12 | EPI_ISL_20078619 | EPI4544040 | EPI4544041 | EPI4544039 | EPI4544043 | EPI4544036 | EPI4544042 | EPI4544038 | EPI4544037 |
| A/turkey/France/21P011811/2021 | 2021-10-25 | EPI_ISL_20078618 | EPI4544032 | EPI4544033 | EPI4544031 | EPI4544035 | EPI4544028 | EPI4544034 | EPI4544030 | EPI4544029 |
| A/turkey/France/21P011515/2021 | 2021-10-11 | EPI_ISL_20078617 | EPI4544024 | EPI4544025 | EPI4544023 | EPI4544027 | EPI4544020 | EPI4544026 | EPI4544022 | EPI4544021 |
| A/turkey/France/21P006932/2021 | 2021-02-26 | EPI_ISL_20078616 | EPI4544016 | EPI4544017 | EPI4544015 | EPI4544019 | EPI4544012 | EPI4544018 | EPI4544014 | EPI4544013 |
| A/turkey/France/21P006292/2021 | 2021-02-17 | EPI_ISL_20078599 | EPI4544008 | EPI4544009 | EPI4544007 | EPI4544011 | EPI4544004 | EPI4544010 | EPI4544006 | EPI4544005 |
| A/turkey/France/21P006282/2021 | 2021-02-15 | EPI_ISL_20078598 | EPI4544000 | EPI4544001 | EPI4543999 | EPI4544003 | EPI4543996 | EPI4544002 | EPI4543998 | EPI4543997 |
| A/turkey/France/21P003275/2021 | 2021-01-15 | EPI_ISL_20078597 | EPI4543992 | EPI4543993 | EPI4543991 | EPI4543995 | EPI4543988 | EPI4543994 | EPI4543990 | EPI4543989 |
| A/turkey/France/21P001724/2021 | 2021-01-09 | EPI_ISL_20078596 | EPI4543984 | EPI4543985 | EPI4543983 | EPI4543987 | EPI4543980 | EPI4543986 | EPI4543982 | EPI4543981 |
| A/turkey/France/20P016491/2020 | 2020-11-12 | EPI_ISL_20078595 | EPI4543976 | EPI4543977 | EPI4543975 | EPI4543979 | EPI4543972 | EPI4543978 | EPI4543974 | EPI4543973 |
| A/turkey/France/20P014153/2020 | 2020-09-30 | EPI_ISL_20078594 | EPI4543968 | EPI4543969 | EPI4543967 | EPI4543971 | EPI4543964 | EPI4543970 | EPI4543966 | EPI4543965 |
| A/turkey/France/20P012832/2020 | 2020-08-27 | EPI_ISL_20078593 | EPI4543960 | EPI4543961 | EPI4543959 | EPI4543963 | EPI4543956 | EPI4543962 | EPI4543958 | EPI4543957 |
| A/turkey/France/20P012492/2020 | 2020-08-12 | EPI_ISL_20078592 | EPI4543952 | EPI4543953 | EPI4543951 | EPI4543955 | EPI4543948 | EPI4543954 | EPI4543950 | EPI4543949 |
| A/turkey/France/20P007717/2020 | 2020-06-09 | EPI_ISL_20078591 | EPI4543944 | EPI4543945 | EPI4543943 | EPI4543947 | EPI4543940 | EPI4543946 | EPI4543942 | EPI4543941 |
| A/turkey/France/20P005076/2020 | 2020-04-06 | EPI_ISL_20078590 | EPI4543936 | EPI4543937 | EPI4543935 | EPI4543939 | EPI4543932 | EPI4543938 | EPI4543934 | EPI4543933 |
