# Supplementary material for: Identification of H1N2 influenza viruses in turkeys after spillover from swine and in vitro characterization
Source: Virus Res. 2025 Sep 21;361:199634. doi: 10.1016/j.virusres.2025.199634 (PMC12506539; doi:10.1016/j.virusres.2025.199634)
Supplement: Supplementary file 1 [file mmc1.zip › Supplementary Figure 1.docx]

**Supplementary Figure 1**. Maximum likelihood phylogeny of the 156 swine and turkey influenza H1_av_N2#E genome sequences obtained from PB2 segment.

The two H1_av_N2#E viruses from 2015 that root the tree are not shown. Blue dots: turkey sequences.

**Supplementary Figure 2**. Maximum likelihood phylogeny of the 156 swine and turkey influenza H1_av_N2#E genome sequences obtained from PB1 segment.

The two H1_av_N2#E viruses from 2015 that root the tree are not shown. Blue dots: turkey sequences.

**Supplementary Figure 3**. Maximum likelihood phylogeny of the 156 swine and turkey influenza H1_av_N2#E genome sequences obtained from PA segment.

The two H1_av_N2#E viruses from 2015 that root the tree are not shown. Blue dots: turkey sequences.

**Supplementary Figure 4**. Maximum likelihood phylogeny of the 156 swine and turkey influenza H1_av_N2#E genome sequences obtained from HA segment.

The two H1_av_N2#E viruses from 2015 that root the tree are not shown. Blue dots: turkey sequences.

**Supplementary Figure 5**. Maximum likelihood phylogeny of the 156 swine and turkey influenza H1_av_N2#E genome sequences obtained from NP segment.

The two H1_av_N2#E viruses from 2015 that root the tree are not shown. Blue dots: turkey sequences.

**Supplementary Figure 6**. Maximum likelihood phylogeny of the 156 swine and turkey influenza H1_av_N2#E genome sequences obtained from NA segment.

The two H1_av_N2#E viruses from 2015 that root the tree are not shown. Blue dots: turkey sequences.

**Supplementary Figure 7**. Maximum likelihood phylogeny of the 156 swine and turkey influenza H1_av_N2#E genome sequences obtained from M segment.

The two H1_av_N2#E viruses from 2015 that root the tree are not shown. Blue dots: turkey sequences.

**Supplementary Figure 8**. Maximum likelihood phylogeny of the 156 swine and turkey influenza H1_av_N2#E genome sequences obtained from NS segment.

The two H1_av_N2#E viruses from 2015 that root the tree are not shown. Blue dots: turkey sequences.

**Supplementary Figure 9.** Comparison of amino acid frequency for the three groups on HA antigenic sites (A) and NA antigenic sites (B). Group 1: Swine sequences. Group 2: Other turkey sequences, outside the turkey cluster. Group 3: Turkey cluster sequences. The size of the letters is proportional to the occurrence of the amino acid at a given position.
