# Supplementary figures and images for: Identification of H1N2 influenza viruses in turkeys after spillover from swine and in vitro characterization
Source: Virus Res. 2025 Sep 21;361:199634. doi: 10.1016/j.virusres.2025.199634 (PMC12506539; doi:10.1016/j.virusres.2025.199634)

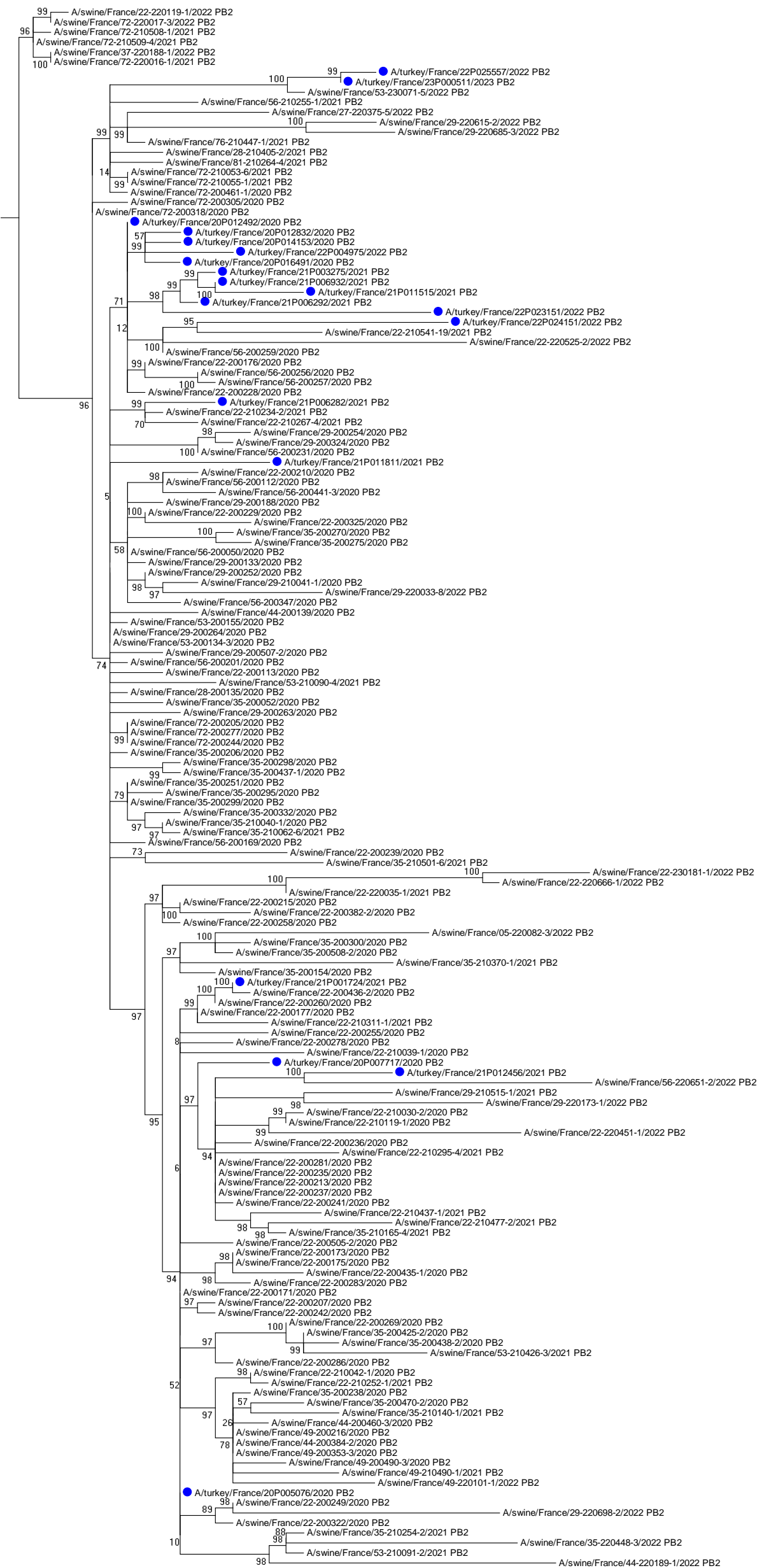

Supplement: Supplementary file 1 [file mmc1.zip › Supplementary_Figure_1.pdf]

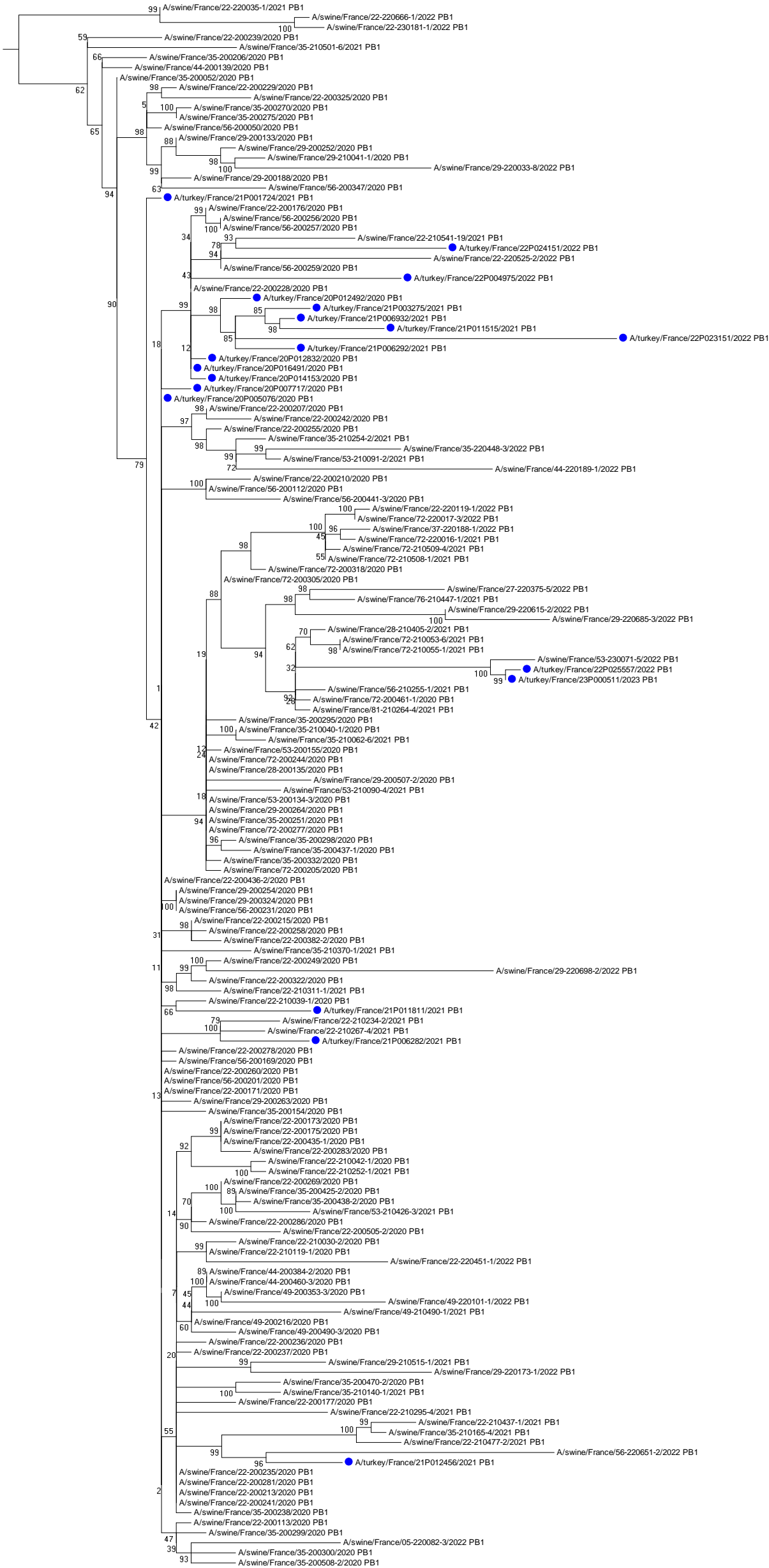

0.001

Supplement: Supplementary file 1 [file mmc1.zip › Supplementary_Figure_2.pdf]

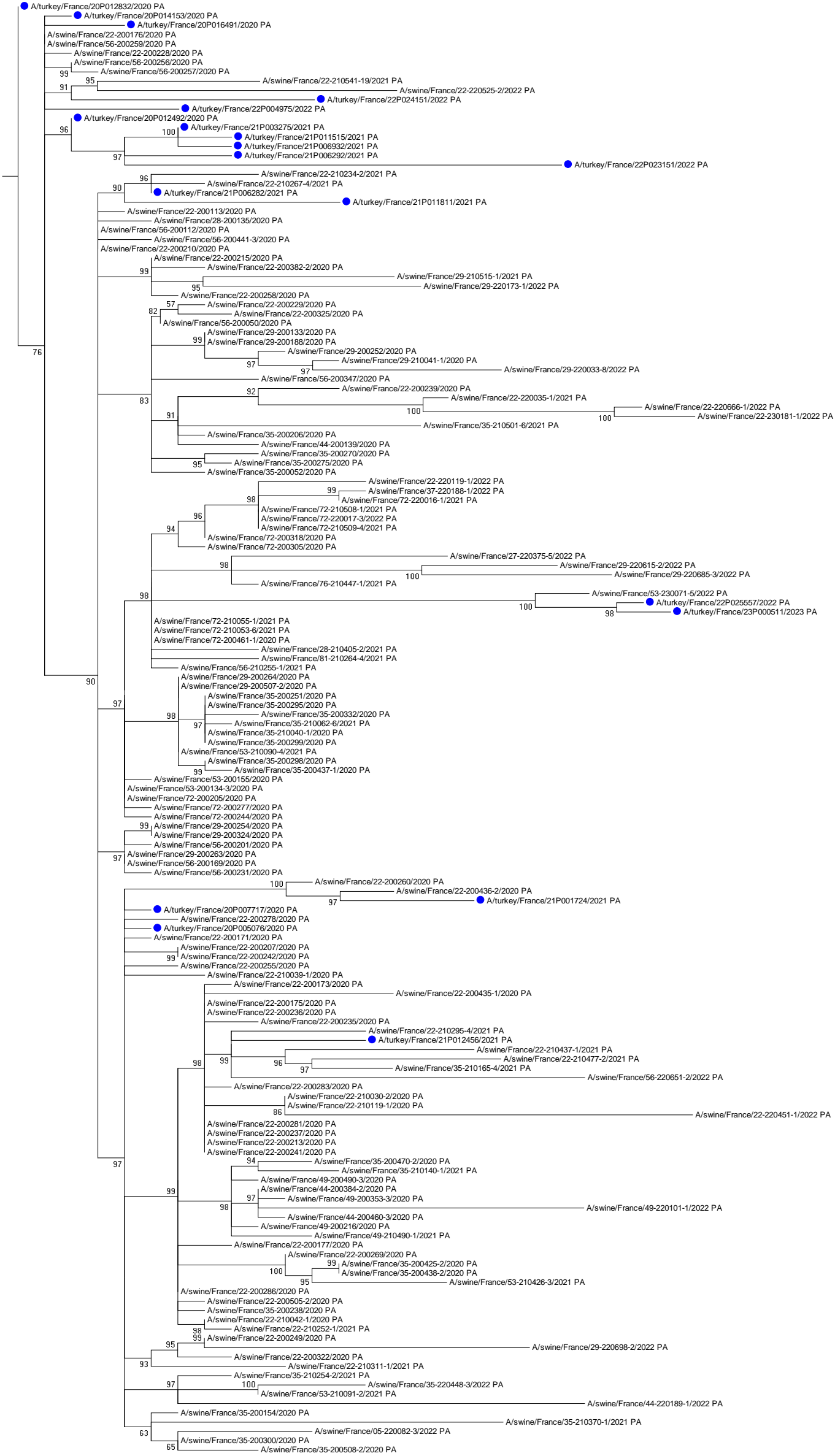

0.0005

Supplement: Supplementary file 1 [file mmc1.zip › Supplementary_Figure_3.pdf]

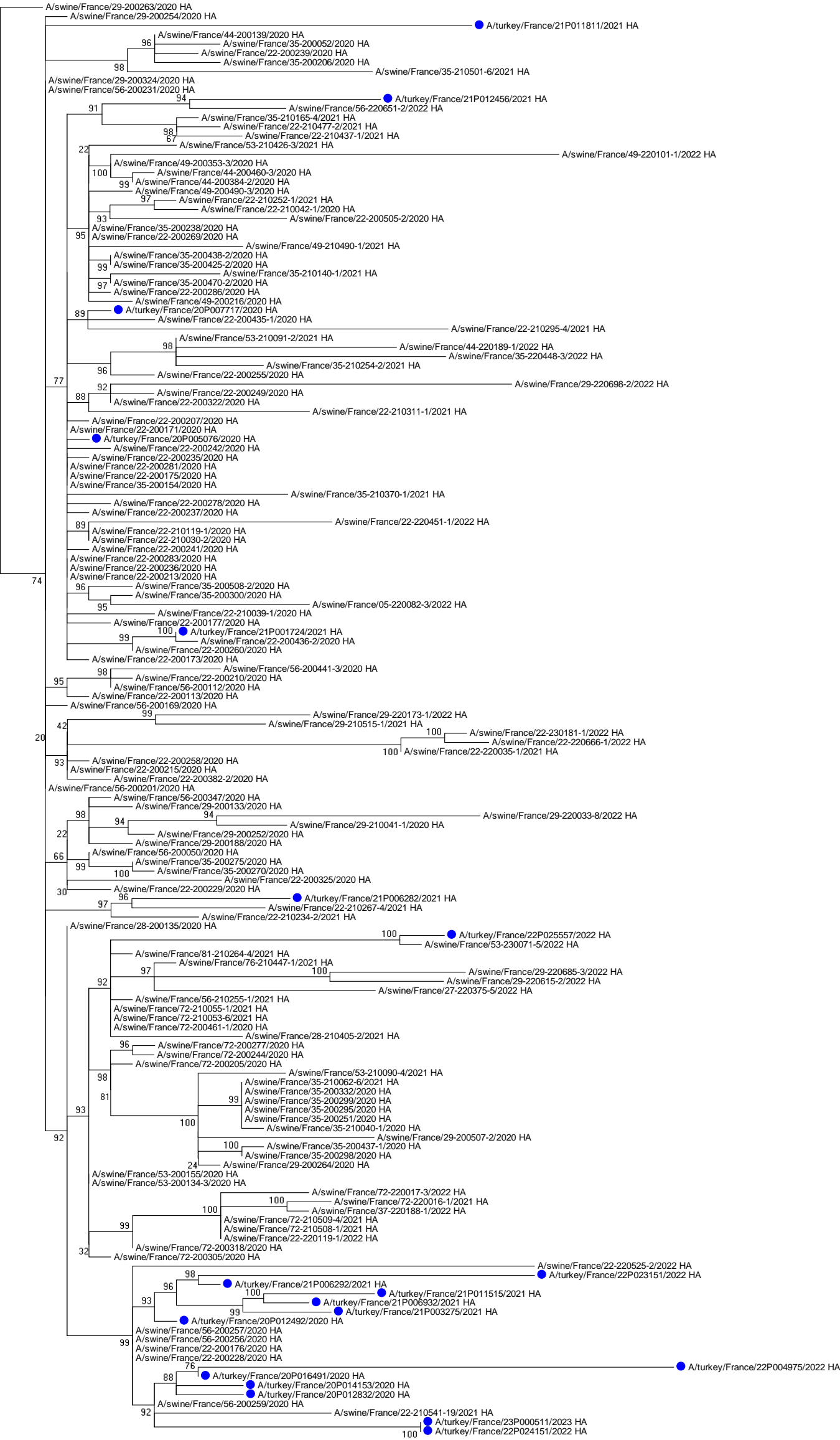

0.0005

Supplement: Supplementary file 1 [file mmc1.zip › Supplementary_Figure_4.pdf]

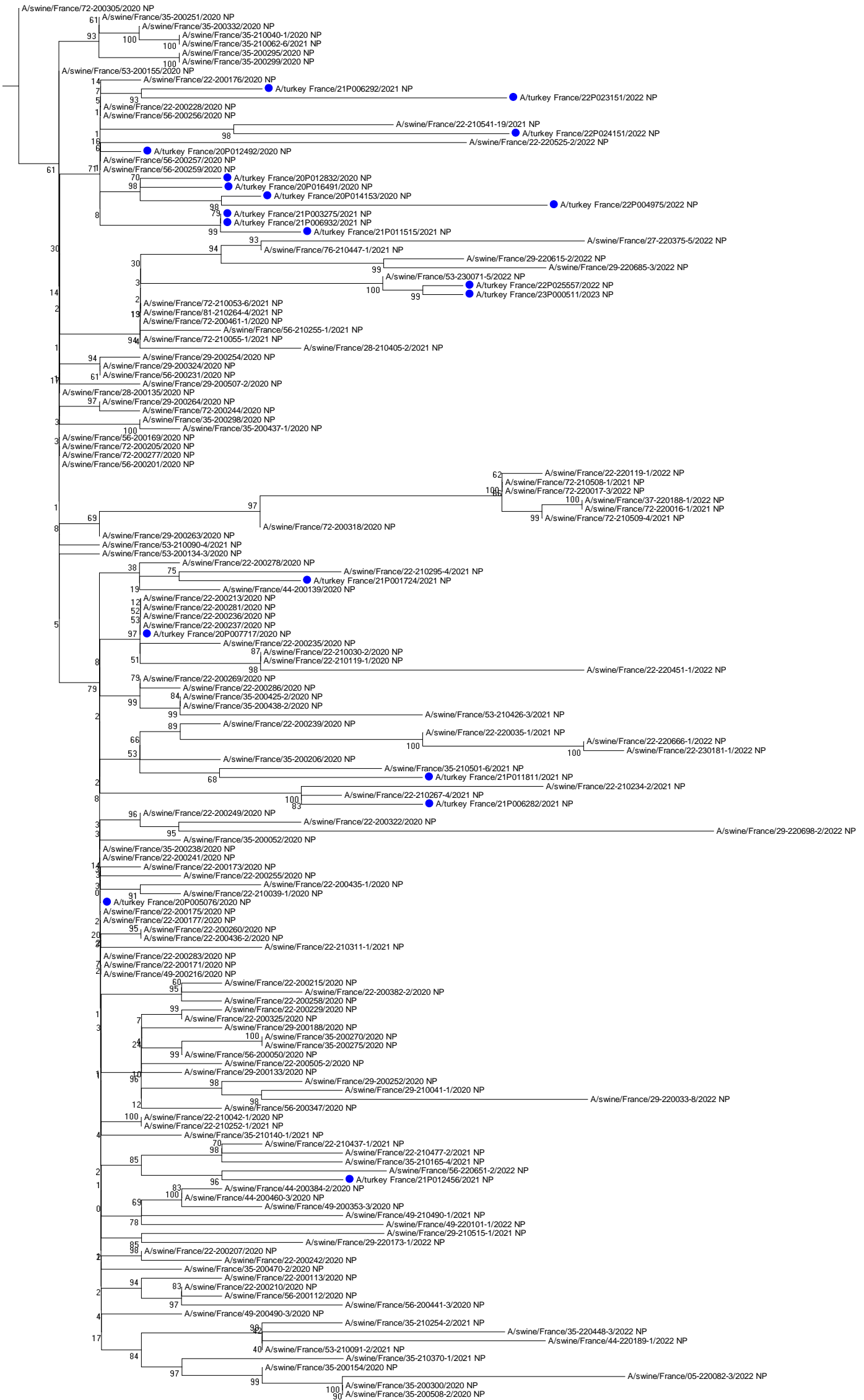

0.0005

Supplement: Supplementary file 1 [file mmc1.zip › Supplementary_Figure_5.pdf]

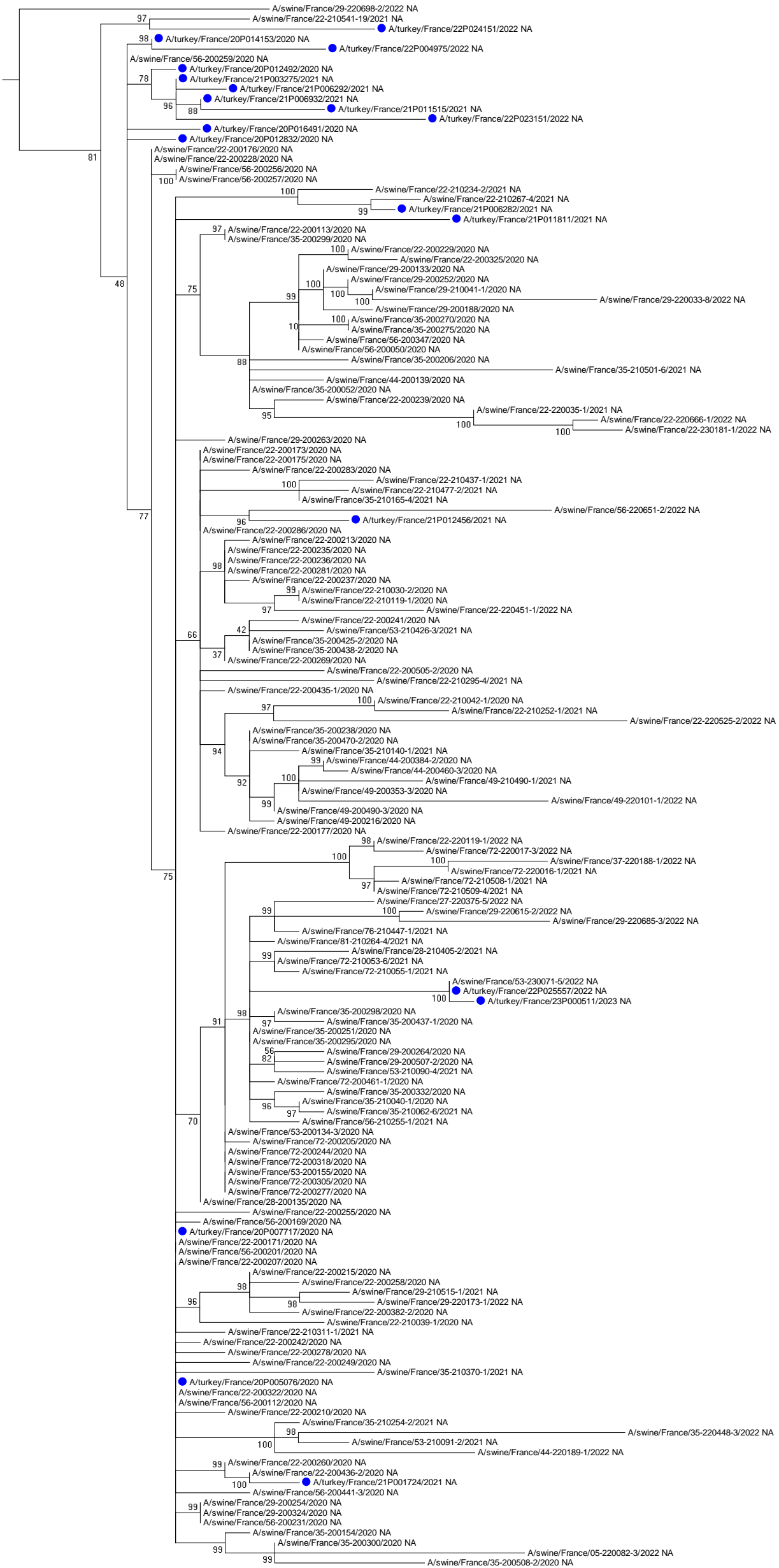

Supplement: Supplementary file 1 [file mmc1.zip › Supplementary_Figure_6.pdf]

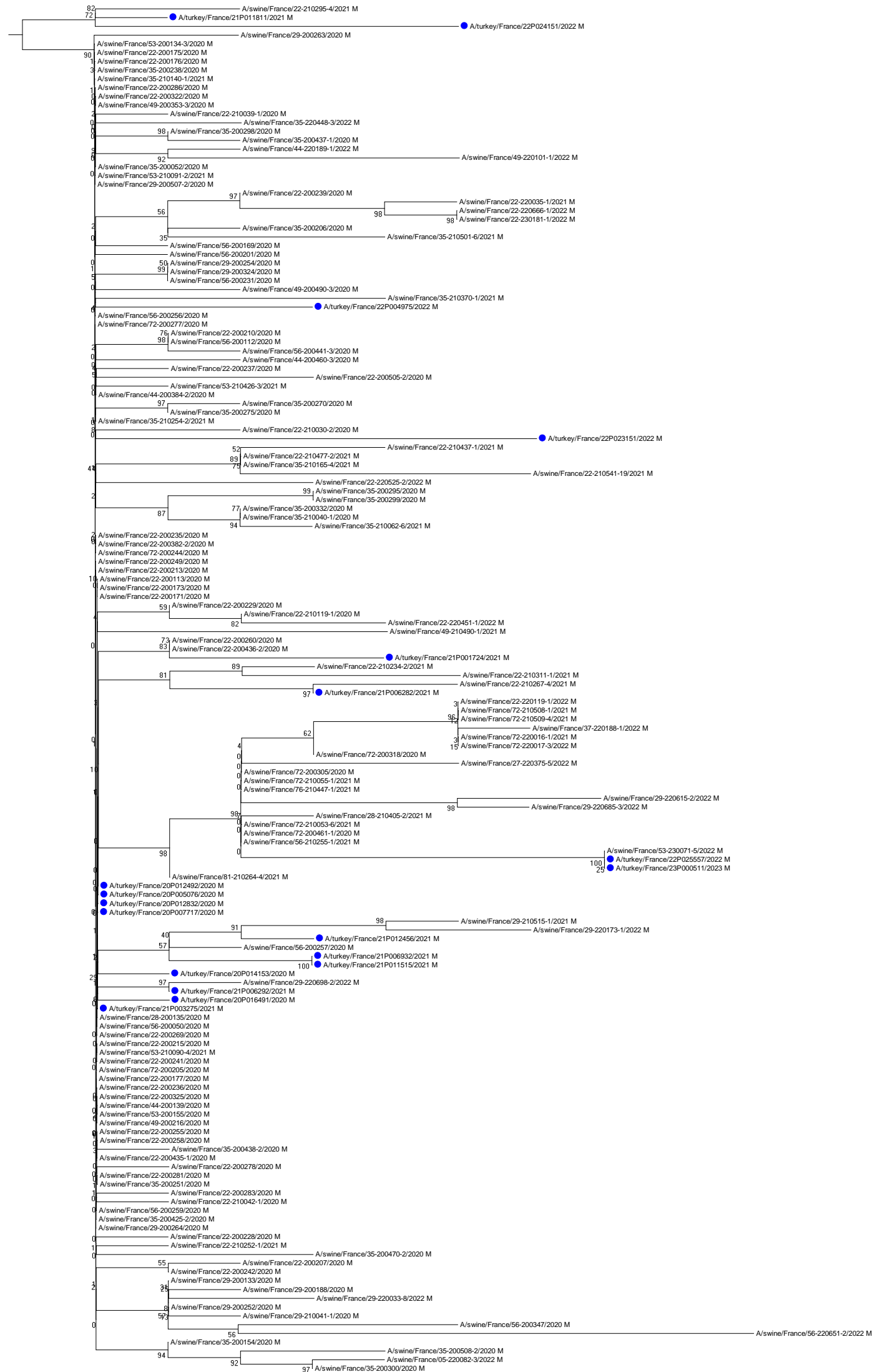

Supplement: Supplementary file 1 [file mmc1.zip › Supplementary_Figure_7.pdf]

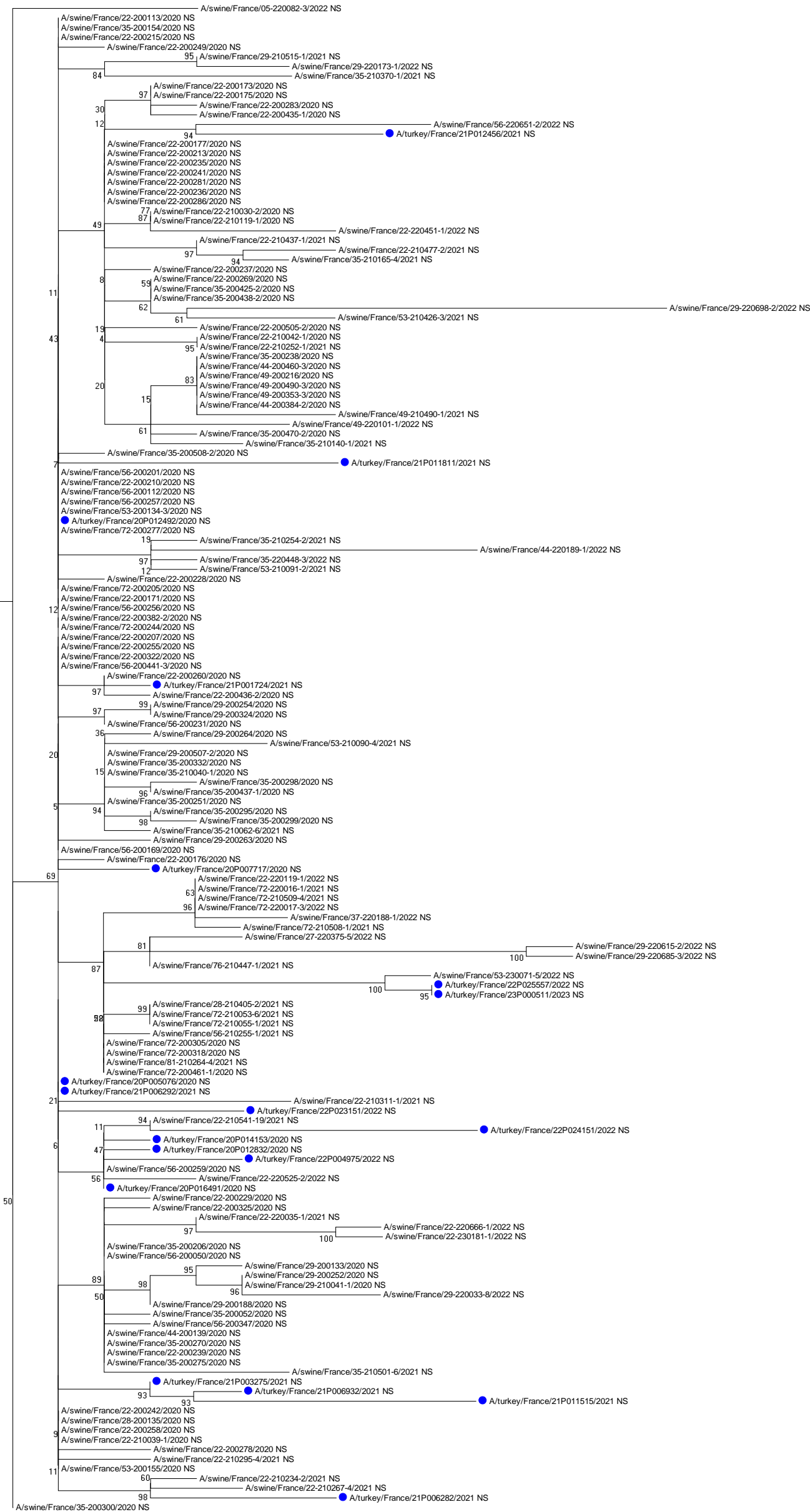

0.001

Supplement: Supplementary file 1 [file mmc1.zip › Supplementary_Figure_8.pdf]
